# Renal developmental genes are differentially regulated after unilateral ureteral obstruction in neonatal and adult mice

**DOI:** 10.1038/s41598-020-76328-3

**Published:** 2020-11-09

**Authors:** Melanie J. Kubik, Maja Wyczanska, Mojca Gasparitsch, Ursula Keller, Stefanie Weber, Franz Schaefer, Bärbel Lange-Sperandio

**Affiliations:** 1 Department of Pediatrics, Ruprecht-Karls-University, Heidelberg, Germany; 2 Division of Pediatric Nephrology, Department of Pediatrics, Dr. v. Hauner Children’s Hospital, University Hospital, Ludwig-Maximilians-University (LMU) Munich, Lindwurmstr.4, 80337 Munich, Germany; 3 University Children’s Hospital, Philipps-University, Marburg, Germany

**Keywords:** Nephrology, Kidney, Kidney diseases

## Abstract

Congenital obstructive nephropathy hinders normal kidney development. The severity and the duration of obstruction determine the compensatory growth of the contralateral, intact opposite kidney. We investigated the regulation of renal developmental genes, that are relevant in congenital anomalies of the kidney and urinary tract (CAKUT) in obstructed and contralateral (intact opposite) kidneys after unilateral ureteral obstruction (UUO) in neonatal and adult mice. Newborn and adult mice were subjected to complete UUO or sham-operation, and were sacrificed 1, 5, 12 and 19 days later. Quantitative RT-PCR was performed in obstructed, intact opposite kidneys and sham controls for *Gdnf*, *Pax2*, *Six4*, *Six2*, *Dach1*, *Eya1*, *Bmp4*, and *Hnf-1β*. Neonatal UUO induced an early and strong upregulation of all genes*.* In contrast, adult UUO kidneys showed a delayed and less pronounced upregulation. Intact opposite kidneys of neonatal mice revealed a strong upregulation of all developmental genes, whereas intact opposite kidneys of adult mice demonstrated only a weak response. Only neonatal mice exhibited an increase in BMP4 protein expression whereas adult kidneys strongly upregulated phosphatidylinositol 3 kinase class III, essential for compensatory hypertrophy. In conclusion, gene regulation differs in neonatal and adult mice with UUO. Repair and compensatory hypertrophy involve different genetic programs in developing and adult obstructed kidneys.

## Introduction

Congenital obstructive nephropathy is a frequent cause of kidney failure in infants and children^1–3^. In contrast to adult kidneys, obstruction in a developing kidney induces severe disruption of nephron maturation and dramatic reduction of functioning nephrons^4,5^. The development of congenital obstructive nephropathy is regulated by a complex interplay of genetic and non-genetic factors^3^. Histologically, congenital obstructive nephropathies are characterized by dysplastic renal growth, interstitial fibrosis and compensatory growth of the intact opposite kidney^6^. Compensatory hypertrophy in the contralateral kidney is different from normal renal growth^7–10^. Two mechanisms are involved in this process: hypertrophy, defined as an increase in nephron size and mass, and hyperplasia, defined as an increase in nephron cell number^9^. The degree of compensatory hypertrophy has been shown to correlate with the extent of obstruction, loss of function in the affected kidney, and level of phosphatidylinositol 3 kinase class III (Pik3c3) expression in the intact opposite kidney of adult mice^7,10–12^.

Several genes are of major importance for renal development and represent candidate genes for congenital anomalies of the kidney and urinary tract (CAKUT). These genes may also play a role in repair, regeneration and compensatory growth after renal injury. We studied the regulation of renal developmental genes that are associated with CAKUT in obstructed, intact opposite and sham-operated kidneys following unilateral ureteral obstruction (UUO) in newborn and adult mice: *Gdnf* (Glial cell line-derived neurotrophic factor), *Pax2* (Paired homeobox gene 2), *Six4 (Sineoculis homeobox homolog 4)*, *Six2 (Sineoculis homeobox homolog 2)*, *Dach1 (Dachshund homolog 1)*, *Eya1 (Eyes absent homolog 1)*, *Bmp4* (Bone morphogenetic protein 4), and *Hnf-1β* (Hepatocyte nuclear factor-1β). Knockout-data for these genes emphasize a possible involvement of these genes with renal malformation^13–15^. Some of these genes may also be involved in repair and regeneration following renal injury. In kidney development, nephron progenitor cells are a transient developmental cell type that gives rise to nephrons. Nephron progenitor cells fully differentiate in mice by postnatal day 5. Many of the genes studied here are only expressed in nephron progenitor cells (*Gdnf*^16^, *Six2*^17^, *Eya1*^18^, *HNF-1ß*^19^ and *Six4*^6^) and are not present in the adult kidney. They were assayed following ureteral obstruction as an upregulation of these genes would indicate a novel repair mechanism involving possible re-activation of the developmental program or de-differentiation of existing cells in the adult kidney.

In kidney development, *Gdnf* promotes the branching and regulates the length of the ureteric bud and further differentiation of the mesenchyme^16,20^. *Gdnf* non-synonymous deleterious variants are reported in about 5% of CAKUT patients^21^*. Pax2* is a key transcriptional factor in nephron specification^22^. Autosomal dominant mutations in the *PAX2* gene cause the renal-coloboma syndrome^23^. It is highly expressed in proliferative tissue^24^ and has anti-apoptotic effects^25,26^. In models of renal ischemia and acute tubular necrosis *Pax2* is re-expressed^27,28^ and required for kidney repair^29^. *Six2* is essential during all stages of kidney development^15,17^. Deficiency in *Six2* during prenatal development is associated with reduced nephron number, chronic renal failure, and hypertension^14^. *SIX2* mutations have been observed in children with renal hypodysplasia^30^. *DACH1* is found in developing human kidneys where it inhibits TGF-β-induced apoptosis^31^. It is a transcription factor that is important for podocyte differentiation and kidney function^32^. *EYA1* is essential for human kidney development^33,34^. *Eya1*^−/−^-knockout-mice show renal agenesis, and mutations in the human *Eya1* gene cause branchio-oto-renal (BOR)-syndrome^34^. *Eya1* interacts strongly with *Six1* and *Six4* to regulate nephron progenitor cells^18^. *Six1/Six4* deficiency leads to kidney and ureter agenesis^35^. *Bmp4,* a member of the TGF-ß family, promotes proliferation, has anti-apoptotic effects and is essential for the outgrowth and positioning of the ureteric bud^36,37^. *Bmp4*^+/−^-knockout-mice show a variety of kidney malformations, ranging from renal hypodysplasia and hydronephrosis to polycystic kidneys^36–38^. BMP4 mutations have been reported in children with renal hypodysplasia^30,39,40^. *Hnf-1β* regulates gene expression in kidney, liver and other epithelial organs^19,41^. During embryogenesis, *Hnf-1β* is required for initiation of nephrogenesis, ureteric bud branching, and nephron segmentation^42^. Dominant-negative expression of *Hnf-1β* leads to cyst formation and hydroureters^25^. Mutations of *HNF-1β* in humans produce maturity-onset diabetes of the young type 5 (MODY5) and cystic abnormalities of the kidney (renal cyst and diabetes-syndrome, RCAD-syndrome).

Several animal models have shown that renal malformations can be caused by obstruction of the urinary tract^1,43,44^. Neonatal UUO interferes with normal nephrogenesis and branching morphogenesis. In order to study the effect of an early obstruction during kidney development on the regulation of these genes in the obstructed kidneys as well as the intact opposite kidneys, we performed unilateral ureteral obstruction (UUO) in newborn and adult mice. In humans, nephrogenesis is completed before birth, but in mice, it continues for 1–2 weeks after birth. Therefore, neonatal UUO at the second day of life serves as a model to study the effects of urinary tract obstruction on renal development^1,45^. In this study, we demonstrate that renal developmental gene regulation differs in neonatal and adult mice upon ureteral obstruction. Our findings support differential regeneration and repair processes in neonatal versus adult mice with UUO.

## Materials and methods

### Experimental protocol

Two-day-old WT mice (C57/BL6) and 7–8 week old adult mice were subjected to complete left ureteral obstruction or sham operation under general anesthesia with isoflurane (3–5% v/v) and oxygen (0.5 L/min) on the second day of life, and at 7–8 weeks, respectively, as described before^46^. The sex distribution was in both groups equal. After recovery, neonatal mice were returned to their mothers until sacrifice 1, 5, 12 and 19 days after surgery (n = 16 per group). The adult mice were also sacrificed 1, 5, 12 and 19 days after surgery (n = 16 per group). All experiments were performed according to national animal protection laws and the guidelines of animal experimentation established and approved by the Regierungspräsidium Karlsruhe and the Committee for Animal Experimentation of the University of Heidelberg (Az 35–9185.81/G-89/05).

### Real-time quantitative reverse transcription-polymerase chain reaction (RT-PCR)

Kidneys from neonatal and adult mice were harvested 1, 5, 12, and 19 days after UUO or sham-operation (n = 4 in each group). Using Quiagen, RNA was isolated (Sigma, Munich) and checked for integrity. RNA was quantified photometrically. Real time RT-PCR was performed using oligo(dT)/random hexamer primers (10:1). One microgram of RNA was reverse transcribed using the Abi Prism 7000 Real Time PCR system (Applied Biosystems, Darmstadt) with specific primers for *18S*, mouse-*Gdnf*, -*Pax2*, -*Six4*, -*Six2*, -*Dach1*, -*Eya1*, -*Bmp4*, and -*Hnf-1β* (Table 1), and Universal Mastermix (Applied Biosystems) with SYBR green to measure PCR products. All the primer pairs have been purchased from Applied Biosystems. We used serial dilutions of an arbitrary cDNA pool to generate a standard curve. Levels of mRNA were normalized to corresponding *18S* quantities measured within the same run. Relative amount of mRNA was calculated using comparative Ct (ΔCt) method.

**Table 1:** Real time RT-PCR primer sequences.

Gene	Forward primer	Reverse primer
18S	AGTTGGTGGAGCGATTTGTC	GCTGAGCCAGTTCAGTGTAGC
Gdnf	CGCTGACCAGTGACTCCAATAT	TTCAGTCTTTTAATGGTGGCTTGA
Pax2	CGAGGAAGTCGAGGTATACACTGA	GCAGGTGCTTCCGCAAAC
Six 4	CAGCTTCACAAGGTAATCTTTCAGTTAC	AGGAACGGTGTATACCACTGCA
Eya1	GAATTTCCTCCTATGGTGCATTGT	GGTAGCTGTACGGTGCCTGTC
Hnf-1β	AGATGTCAGGAGTGCGCTACAA	CTGGTCACCATGGCACTGTTA
Six2	ACCACGCAAGTCAGCAACTG	TTGTGGCTGCTGGAATTGG
Dach1	GTTGGCAGCAGTGGTGGTT	CAGATGGTTGAGAGGATGGCTAA
Bmp4	GTGAGGAGTTTCCATCACGAAGAA	GGATGCTGCTGAGGTTGAAGAG

### Identification of proliferation and BMP4

Proliferation and BMP4 expression were examined by immunohistochemistry as described previously^45^. Formalin-fixed, paraffin-embedded sections of UUO-, IO- and sham-operated kidneys of newborn and adult mice (n = 8 per time point) were subjected to antigen retrieval and incubated with Ki67 antibody (DAKO M7248) to stain proliferating nuclei, or with BMP4 antibody (ab 6296, Abcam). Control sections were stained simultaneously with a non-specific, species-controlled primary antibody. We used biotinylated goat anti-rat IgG (Southern Biotechnology Associates, Inc., Birmingham, AL) as secondary antibody. After incubation with ABC reagent (Vectastain, Vector Laboratories, Burlingame, CA) and counterstaining with hemalaun, proliferation was calculated by counting the number of Ki67 positive cells in 20 sequentially selected microscopic high power fields (hpfs) at × 400 magnification. Proliferation was expressed as the mean number of Ki67 positive cells per hpf. Photomicrographs of proliferating cells and BMP4 expression in the obstructed and intact opposite kidney of adult and newborn mice are shown in Fig. 5.

### Detection of cellular apoptosis

Apoptotic cells were detected by the terminal deoxynucleotidyl transferase (TdT)- mediated dUTP-biotin nick end labeling (TUNEL) assay as described previously^4^. Briefly, formalin-fixed tissue sections were de-paraffinized and rehydrated followed by incubation with proteinase K (20 µg/ml). After quenching, equilibration buffer was applied, followed by working strength enzyme (ApopTag Peroxidase In Situ Apoptosis Detection Kit, Millipore, Schwalbach, Germany). Cells were regarded as TUNEL positive if their nuclei were stained black and displayed typical apoptotic morphology. Apoptosis was calculated in neonatal mice (n = 8 per time point) by counting the number of TUNEL positive cells in 20 sequentially selected microscopic fields of view (hpfs) at × 400 magnification and expressed as the mean number of cells per hpf.

### Western immunoblotting

Kidneys of UUO and control mice were harvested on 1, 5, 12 and 19 days after obstruction (n = 3 in each group) as described previously^4^. In brief, kidneys were homogenized in protein lysis buffer (Triton-X 100 1%, Tris 100 mM, Na_4_P_2_O_3_ 100 mM, NaF 100 mM, EDTA 10 mM) containing a cocktail of proteinase and phosphatase inhibitors (1 mM Na_3_VO_4_, 1 mM PMSF, 10 µg/ml leupeptin, 10 µg/ml aprotinin) and centrifuged for 60 min at 20,000×*g*. The protein content of the supernatants was measured using the BCA Protein Assay Kit (Pierce #23225). 15–20 µg of protein were separated on polyacrylamide gels at 180 V for 45 min and blotted onto nitrocellulose membranes (105 V, 80 min). After blocking antibody-specific for 2 h in Tris-buffered saline with Tween-20 containing 5% nonfat dry milk and/or BSA, blots were incubated with primary antibodies 2 h at room temperature or at 4 °C overnight. Gdnf antibody (Abcam plc, Cambridge, UK, ab 17732; 1:1000), BMP4 antibody (clone V9, Sigma, Germany, V-6630; 1:200), Pik3c3 antibody (Echelon Biosciences, Z-R016, 1:100) were used for western blot analysis. GAPDH (DUNN Labortechnik H86540M) was used as an internal loading control and to normalize samples. Blots were washed with Tris-buffered saline with Tween-20 and incubated with horseradish peroxidase-conjugated secondary antibody for 1 h at room temperature. Immune complexes were detected using enhanced chemiluminescence method. Blots were exposed to x-ray films (Kodak, Stuttgart, Germany), the films were scanned and protein bands were quantified using the densitometry program Image J. Each band represents one single mouse kidney.

### Statistical analysis

Data are presented as mean ± standard error. Comparisons between groups were made using one-way analysis of variance followed by the Student–Newman–Keuls test. Comparisons between left and right kidneys were performed using the Students t-test for paired data. Statistical significance was defined as *p* < 0.05.

## Results

### Gene regulation differs in neonatal and adult kidneys

**Figure 1:**
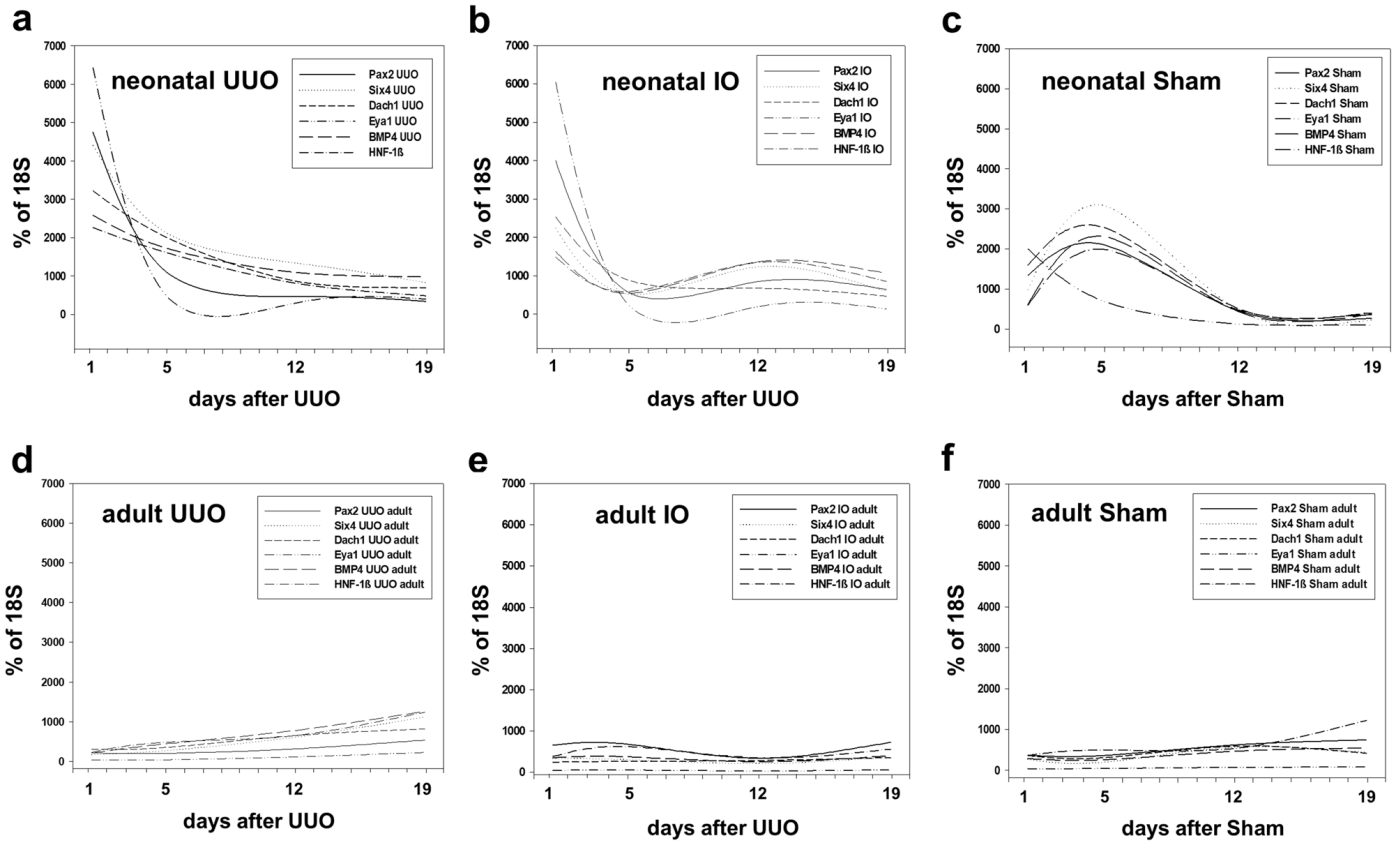
Differential renal developmental gene expression in neonatal and adult mouse kidneys after unilateral ureteral obstruction (UUO). Quantitative RT-PCR of renal developmental genes in neonatal and adult kidneys of WT mice 1, 5, 12, and 19 days after UUO. Surgery was performed in neonatal mice on the second day of life (**a**–**c**) and in adult mice at the age of 7–8 weeks (**d**–**f**). A second scale that shows the early postnatal age (d1–d7 after surgery) is in supplementary material (Supplementary Figure S10) online. IO = intact opposite kidney, Sham = sham-operated kidney.

**Figure 2:**
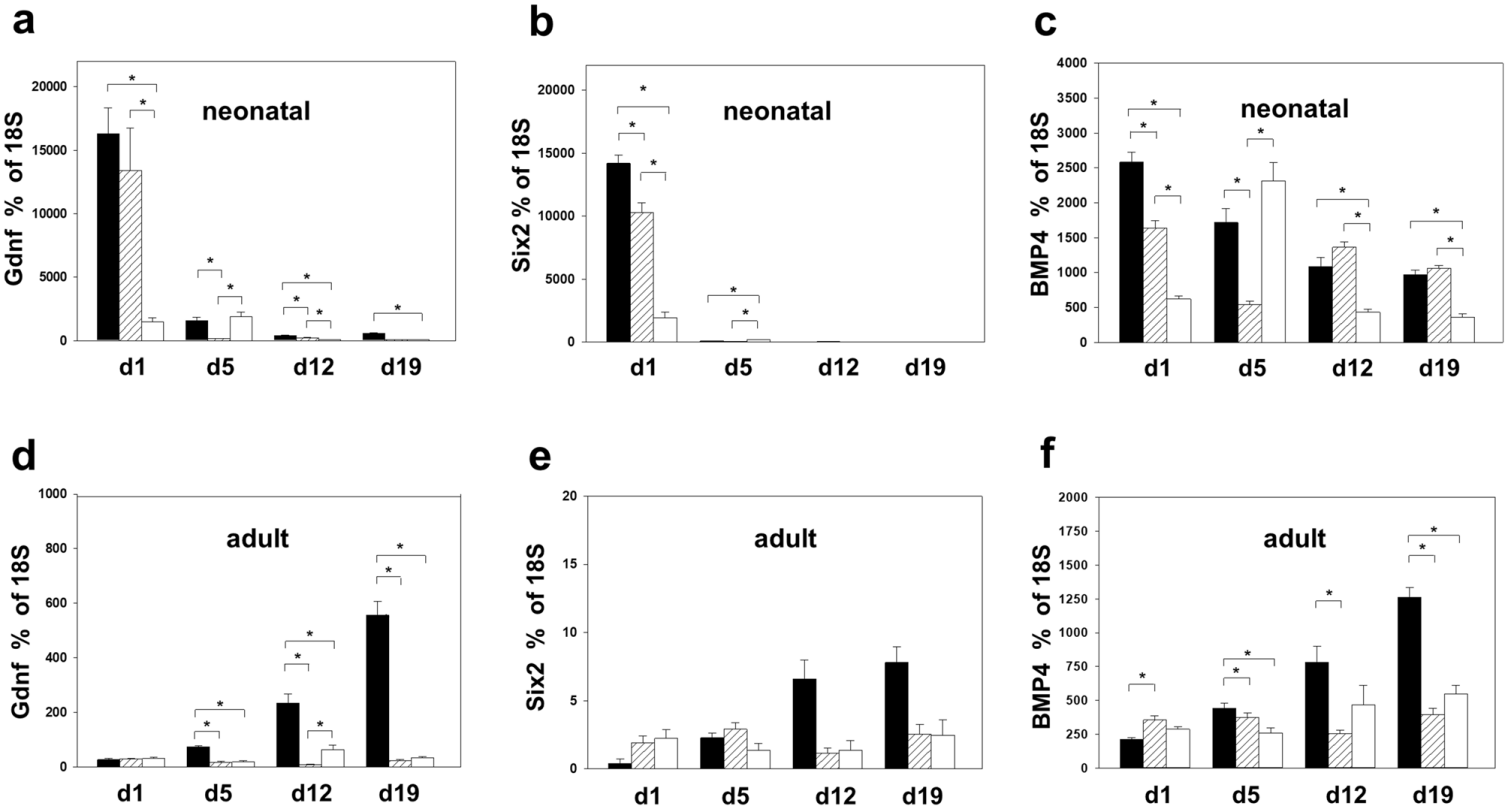
*Gdnf, Six2* and *BMP4 mRNA message* in neonatal and adult mouse kidneys after UUO. *Gdnf, Six2* and *BMP4* gene expression were measured in UUO-, intact opposite (IO)-, and sham-operated kidneys of neonatal (**a**–**c**) and adult (**d**–**f**) mice by quantitative RT-PCR. Gdnf showed the highest upregulation after UUO, followed by Six2. Neonatal but not adult intact opposite kidneys upregulated *BMP4* mRNA. Additional graphs for *Pax2*, *Six4*, *Dach1*, *HNF-1ß* and *Eya1* are in supplementary material (Supplementary Figure S11 and S12) online. UUO = obstructed kidneys (black), IO = intact opposite kidneys (striped), sham = sham operated (white). n = 4; *p < 0.05.

Developmental genes were analyzed in neonatal and adult kidneys with unilateral ureteral obstruction (UUO) at 1, 5, 12, and 19 days after surgery (n = 4 in each group) (Figs. 1 and 2). Quantitative RT-PCR for *Pax2*, *Six4*, *Dach1*, *Eya1*, *Bmp4, Hnf-1β, Gdnf, and Six2* was performed in obstructed (UUO), intact opposite (IO), and sham-operated kidneys (Sham). UUO resulted in a significant mRNA-upregulation of all developmental genes in neonatal obstructed and intact opposite kidneys compared to sham operated controls (Figs. 1a–c and 2a–c). This upregulation was strongest directly after UUO was performed (d1) and decreased toward day 19. Adult kidneys showed a weaker gene expression than neonatal kidneys with a delayed upregulation (d12, d19) in obstructed kidneys, and transient upregulation of only *Pax2* at d1 and d5 in intact opposite kidneys compared to sham operated controls (Fig. 1d–f). In neonatal obstructed kidneys *Gdnf* showed the highest mRNA-expression (Fig. 2a), followed closely by *Six2* (Fig. 2b). Neonatal contralateral (IO) kidneys showed a parallel expression pattern with initial upregulation of all genes on day 1 (Fig. 2a,b). Only *Bmp4* mRNA showed an early upregulation in intact opposite kidneys of neonatal mice following UUO (Fig. 2c). This *Bmp4* upregulation in IO-kidneys remained significant 12 and 19 days after obstruction (n = 4 in each group) (Fig. 2c). Adult obstructed kidneys showed no initial upregulation on day 1 after UUO (Fig. 2d–f). Particular genes showed increased expression beginning on day 12: *Gdnf* and *Bmp4* (Fig. 2d,f). By contrast, *Six2* was not present in the adult kidney. Intact opposite kidneys of adult mice showed no *Bmp4* upregulation from d1 to day 19 after surgery (Fig. 2f).

### GDNF and BMP4 protein expression after UUO. BMP4 expression increases only in neonatal but not in adult intact opposite kidneys

**Figure 3:**
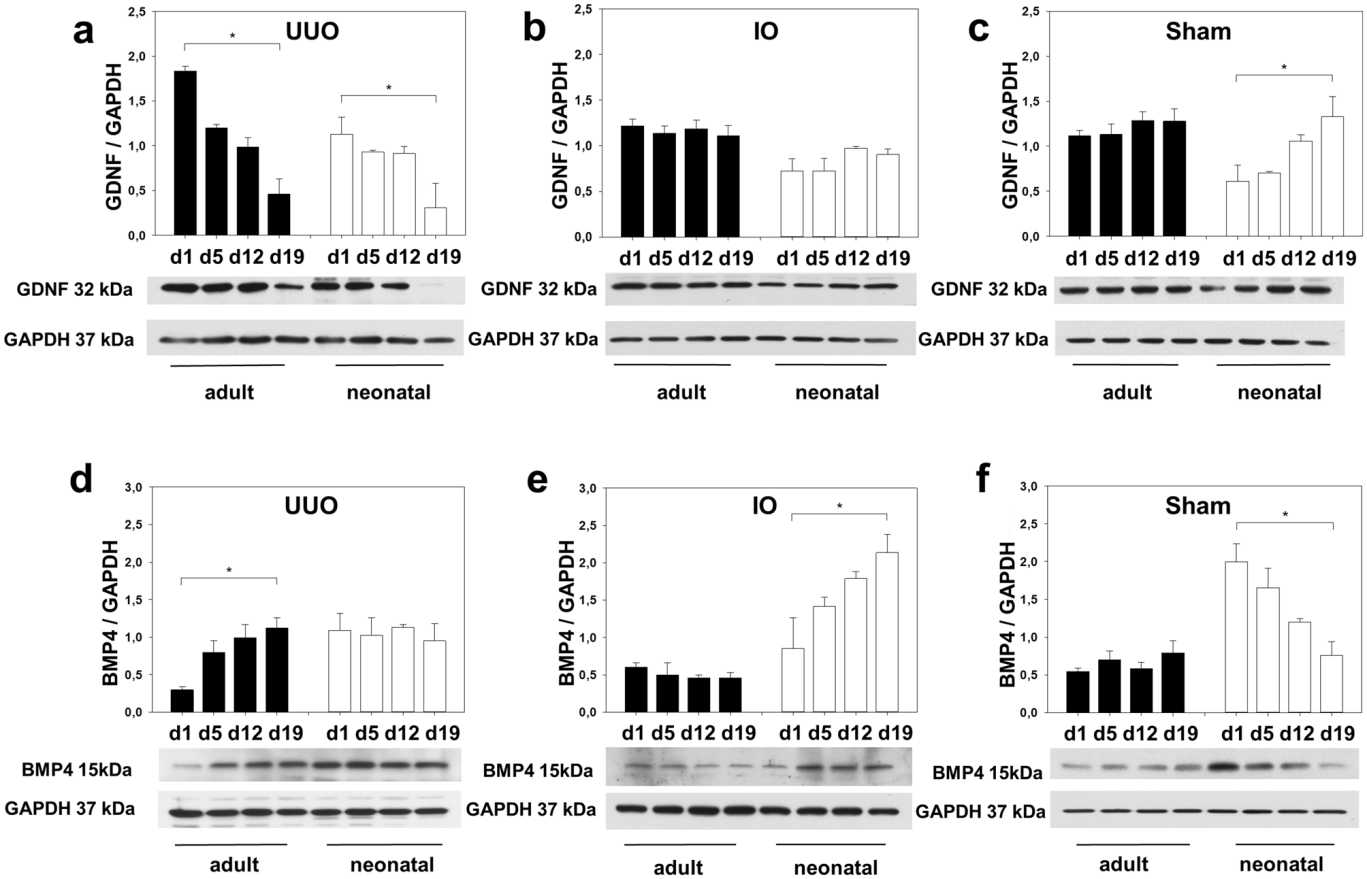
Gdnf expression decreased after UUO and BMP4 increased in neonatal intact opposite kidneys with compensatory renal growth. Neonatal mice (white bars) and adult mice (black bars) were subjected to UUO or sham operation. Whole kidneys were processed for western blot analysis at day 1, 5, 12, and 19 after surgery. Gdnf was reduced in both neonatal and adult UUO-kidneys (**a**). Gdnf expression did not change in intact opposite kidneys (**b**), but increased in sham-operated neonatal kidneys (**c**). BMP4 expression increased in adult UUO-kidneys (**d**) and in neonatal intact opposite kidneys (**e**) after obstruction. BMP4 decreased in neonatal sham-operated controls (**f**). The shown western blot images are cropped, for uncropped western blots see Supplementary Fig. S1–S6 online. For details on significance between groups see Supplementary Table S1 online. n = 3; *p < 0.05.

We next questioned whether the difference in *Gdnf* and *Bmp4* message following UUO was mirrored by the GDNF and BMP4 protein expression in the obstructed, intact opposite, and sham-operated kidney of newborn and adult mice using Western blot (n = 3 in each group). As shown in Fig. 3a, GDNF expression markedly decreased in both adult and neonatal mice following UUO. Similarly, GDNF abundance was equal in IO- and sham operated kidneys of neonatal and adult mice (Fig. 3b,c). In contrast BMP4 expression was markedly upregulated in adult UUO-kidneys following obstruction but did not change in neonatal kidneys with UUO (Fig. 3d). In intact opposite kidneys of neonatal mice BMP4 was highly expressed (Fig. 3e). In contrast, BMP4 did not change in adult IO-kidneys after surgery. Sham operated kidneys of neonatal mice showed a marked decrease in BMP4 expression (Fig. 3f). These results suggest that BMP4 may be involved in compensatory growth in neonatal IO-kidneys.

### Kidney to body weight ratio indicates compensatory growth of neonatal IO-kidneys

**Figure 4:**
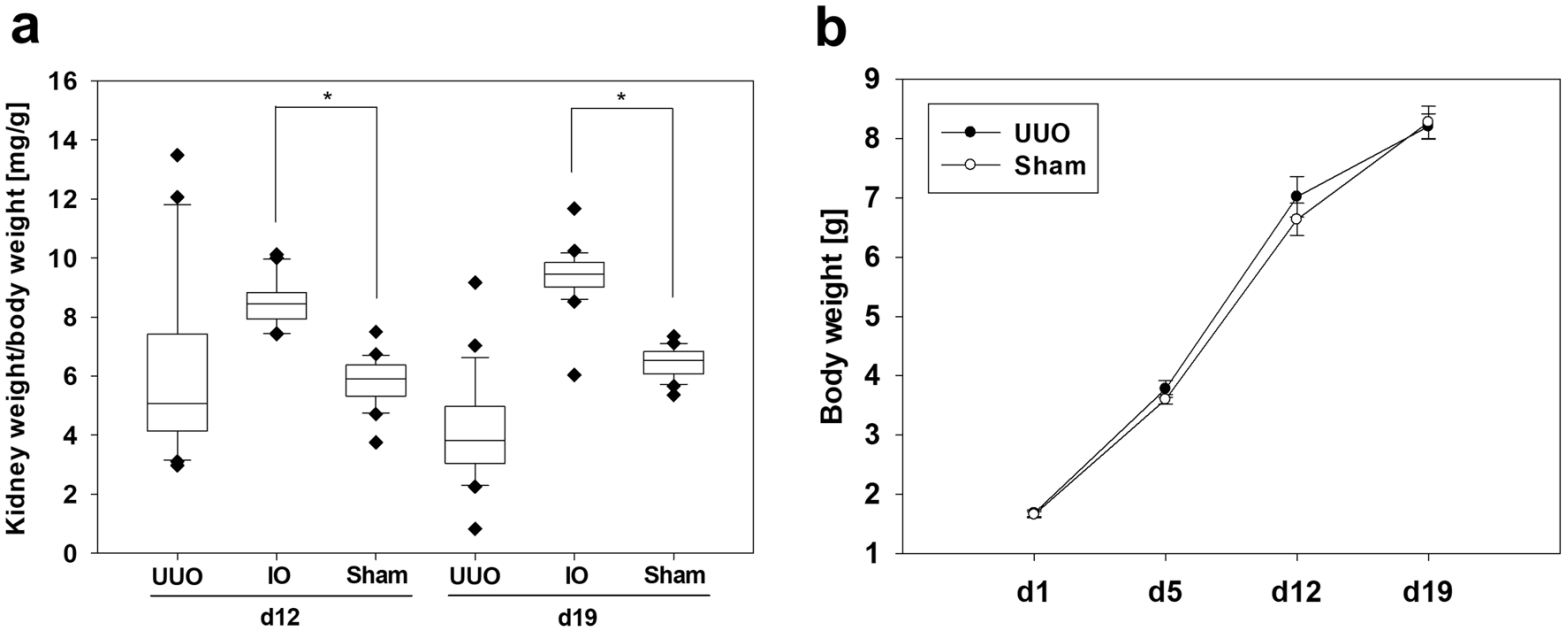
Kidney weight of neonatal intact opposite kidneys increased after UUO. Kidney weight/body weight ratio [mg/g] from neonatal mice after 12 and 19 days of unilateral ureteral obstruction (UUO) (**a**). The weight of the intact opposite kidney (IO) was on average significantly higher than the weight of the sham-operated kidney. There was no difference in the body weight gain between mice with UUO or sham operation (**b**). UUO/IO n = 20, sham n = 20; *p < 0.05.

The kidney and body weights of neonatal mice were measured on days 12 and 19 after surgery (n = 20 per group) (Fig. 4a). The kidney weight/body weight ratio in mg/g was calculated. The kidney weight/body weight ratios of intact opposite (IO) kidneys were significantly higher than of the sham-operated kidneys on both days after surgery. UUO did not cause any differences in body weight of mice compared to the sham-operated controls (Fig. 4b). These data confirm the compensatory growth of the intact opposite kidney.

### Proliferation, apoptosis and localization of BMP4

**Figure 5:**
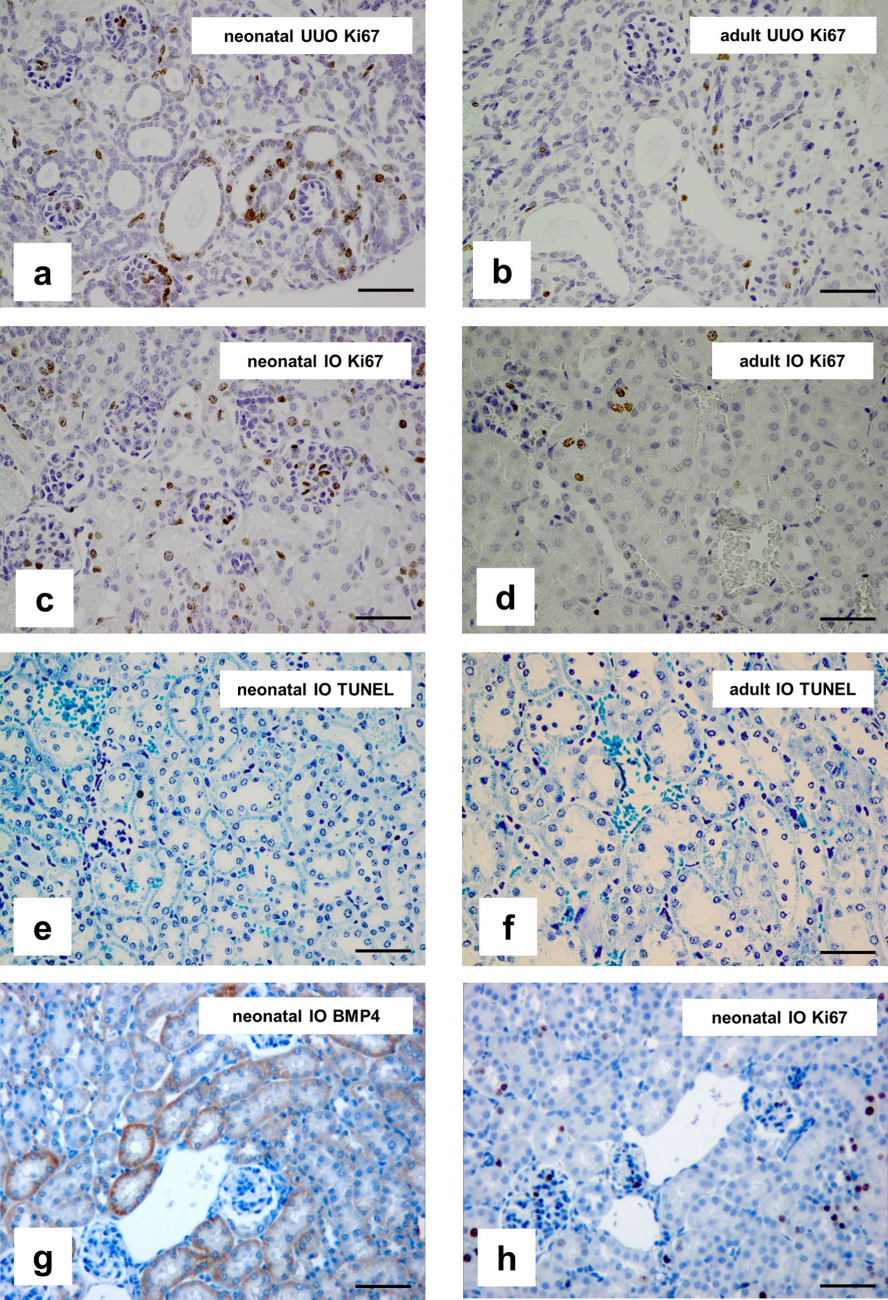
Proliferation is higher in neonatal kidneys and does not co-localize to BMP4. Immunohistochemical staining of Ki67 in neonatal UUO kidneys (**a**), adult UUO kidneys (**b**), neonatal intact opposite kidneys (**c**), and adult intact opposite kidneys (**d**) 5 days after surgery. Dark brown nuclei indicate Ki67 positive proliferating cells. TUNEL staining for renal apoptosis in neonatal intact opposite kidneys (**e**) and adult intact opposite kidneys (**f**) 19 days after surgery. BMP4 is expressed in tubular cells of neonatal intact opposite kidneys (**g**) and does not co-localize to proliferating cells (**h**). A photograph of an obstructed (UUO) kidney at day 5 after ureteral obstruction is in supplementary material (Supplementary Figure S13) online. Staining of sham-operated controls are in supplementary material (Supplementary Figure S14) online. Bar = 100 μm. Magnification of 200 ×.

Proliferation of tubular, glomerular, and interstitial cells was studied in newborn and adult mice kidneys following UUO using Ki67 immunostaining (Fig. 5a–d). Proliferation was higher in neonatal UUO kidneys (Fig. 5a) and neonatal intact opposite kidneys (Fig. 5c) than in adult UUO kidneys (Fig. 5b) and adult intact opposite kidneys, respectively (Fig. 5d). Apoptosis was hardly detectable in kidneys with compensatory growth (Fig. 5e,f). BMP4 expression localized to proximal and distal tubular structures in intact opposite kidneys of neonatal mice (Fig. 5g). No co-localization of BMP4 expression and Ki67 positive cells in intact opposite kidneys of neonatal mice was observed, suggesting that BMP4 may induce nephron hypertrophy without stimulating proliferation (Fig. 5g,h).

### Proliferation and apoptosis in neonatal kidneys after UUO

**Figure 6:**
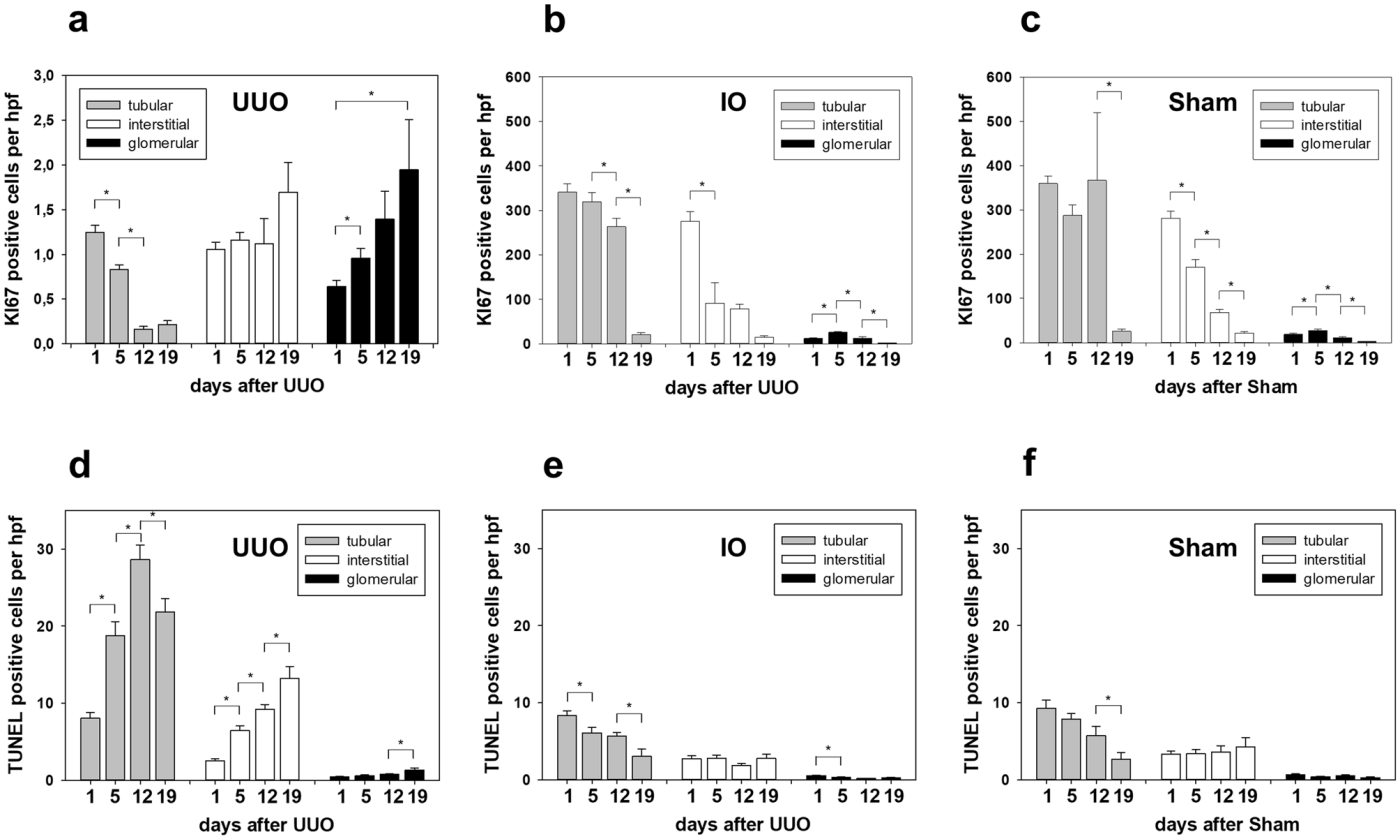
Proliferation and apoptosis in neonatal kidneys after UUO. Renal sections of UUO-, IO- and sham-operated kidneys of neonatal mice were stained for tubular (gray bars), interstitial (white bars) and glomerular (black bars) proliferation (Ki67 antibody) (**a**–**c**) and apoptosis (TUNEL) (**d**–**f**) at 1, 5, 12 and 19 days after surgery and analyzed in 20 high-power fields (hpf) per section at × 400. For details on significance between groups see Supplementary Table S2 online. n = 8; *p < 0.05.

Proliferation and apoptosis of tubular, interstitial and glomerular cells were analyzed using Ki67 and TUNEL staining in obstructed (UUO), intact opposite (IO) and sham-operated (Sham) kidneys (Fig. 6). Following obstruction, proliferation decreased significantly in tubular cells of UUO kidneys (Fig. 6a). In contrast, proliferation of tubular cells in the intact opposite kidney was 300 × times higher than in obstructed neonatal kidneys (Fig. 6b). Sham operated controls indicated the highly proliferative status of the developmental neonatal kidney (Fig. 6c). Apoptosis increased significantly in the neonatal kidney with UUO (Fig. 6d). By contrast, apoptosis was low in intact opposite kidneys (Fig. 6e) and sham operated controls (Fig. 6f).

### Pik3c3 expression

**Figure 7:**
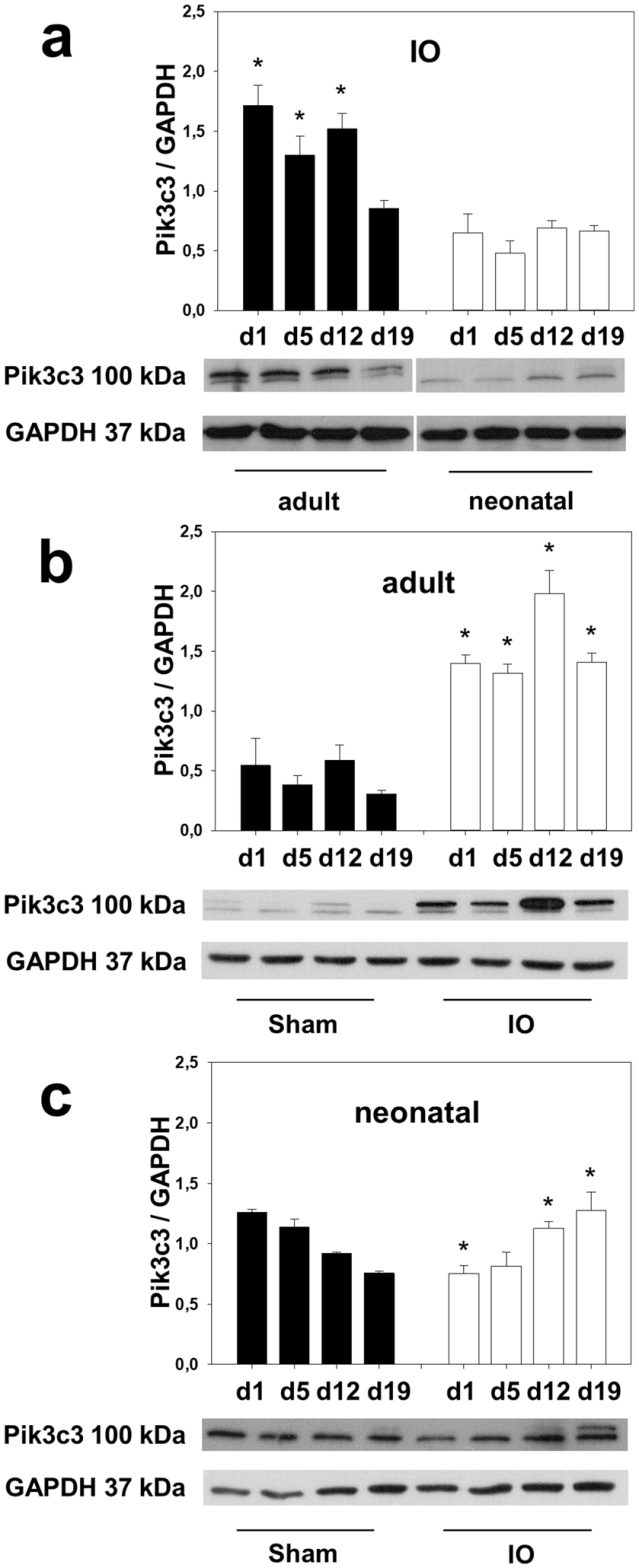
Phosphatidylinositol 3 kinase class III (Pik3c3) expression is upregulated in intact opposite kidneys. Neonatal and adult mice were subjected to UUO or sham operation. Whole kidneys were processed for western blot analysis at day 1, 5, 12, and 19 after surgery. Pik3c3 expression, which controls compensatory hypertrophy, was strongly upregulated in adult intact opposite kidneys (**a** and **b**) and less in neonatal IO-kidneys (**a** and **c**). The shown western blot images are cropped, for uncropped western blots see Supplementary Fig. S7–S9 online. For additional blots on Pik3c3 expression in UUO and sham-operated kidneys see Supplementary Figure S15. For details on significance between groups see Supplementary Table S3 online. n = 3; *p < 0.05. Significance markings reflect the differences between groups, compared to each other on the same points in time. The images for (**a**) were rearranged for uniform presentation. The brightness of the western blot images was changed after the analysis for uniform presentation.

We analyzed Pik3c3, which controls compensatory nephron hypertrophy in obstructed (UUO), intact opposite (IO) and sham-operated (Sham) kidneys of neonatal and adult mice at 1, 5, 12, and 19 days after surgery using Western blot (n = 3 in each group) (Fig. 7). Pik3c3 expression increased in the intact opposite kidneys of adult mice following surgery (Fig. 7a,b), suggestive of mediating the compensatory nephron growth in the contralateral kidney. For the first time we could show an upregulation of Pik3c3 in neonatal IO-kidneys, which was less pronounced (Fig. 7a,c), indicating a different regulation of compensatory hypertrophy in neonatal and adult mice after renal injury.

## Discussion

This study addresses the effect of early renal obstruction on the regulation of renal developmental genes (*Gdnf*, *Pax2*, *Six4*, *Six2*, *Eya1, Dach1*, *Bmp4* and *Hnf-1β*) in the obstructed as well as the intact opposite kidneys. In addition, it compares how genes that are associated with CAKUT are regulated in neonatal and adult kidneys after UUO. Our model demonstrated that the regulation of renal developmental genes differs significantly in both the obstructed and the intact opposite kidneys of neonatal versus adult mice. While neonatal mice showed an early upregulation of all developmental genes in both obstructed and intact opposite (IO) kidneys, adult mice exhibited a reversed expression pattern with a delayed and mostly weaker mRNA increase.

In neonatal UUO-kidneys *Gdnf* showed the highest mRNA upregulation followed closely by *Six2*, *Eya1*, *Pax2*, and *Six4*. *Dach1, Bmp4,* and *Hnf-1β* showed a weaker but still significant mRNA increase following UUO in neonatal mice. Neonatal contralateral kidneys demonstrated a parallel expression pattern with initial upregulation of all genes after obstruction. *Gdnf*, *Six2*, *Eya1*, *Pax2* and *Dach1* message was almost as strong in neonatal IO-kidneys as in neonatal UUO-kidneys. This dramatic upregulation of developmental genes in neonatal kidneys shows that nephron progenitor cells are present and stimulated following injury. Postnatal nephrogenesis in mice is tightly regulated^47^; it ceases in the first 5 days after birth and nephron progenitor cells that express developmental genes are no longer present. Our findings are in line with this showing that adult mice with completed nephrogenesis showed a delayed and much weaker gene response with transient upregulation of only *Pax2*. This upregulation of *Pax2* in adult mice probably occurs in association with the proliferative response to renal injury^29^. *Pax2* is a survival factor for renal collecting duct cells and was upregulated in tubular cells after ischemia^28^. Only Pax2 positive tubular cells underwent complete mitosis after kidney injury, making it an important factor for proliferation and repair^47^. PAX2 protein is expressed in collecting duct cells, but also in cells of the connecting tubule and the thick ascending limb, as well as in fibroblasts and proximal tubular cells. Here we show that *Pax2 is* upregulated after UUO, possibly in order to induce regeneration and repair processes. The upregulation of developmental genes is substantially stronger and faster in neonatal than in adult kidneys. While neonatal kidneys react with an immediate upregulation of certain developmental genes as early as 24hours after damage by obstruction, indicating that they may be able to directly stimulate their developmental program, adult kidneys only show partial and weaker upregulation after 2 weeks of persisting damage. This inverse regulation of renal developmental genes implies that regenerative and repair processes are different in neonatal and adult kidneys.

Most of the genes in this study promote proliferation and inhibit apoptosis. *Gdnf,* which showed highest mRNA expression in neonatal UUO and IO-kidneys, may act as a survival factor following obstruction. Gdnf expression has been shown to be upregulated after podocyte injury, and to inhibit apoptosis and promote differentiation^48^. In our study, *Gdnf* mRNA message was upregulated early in obstructed and contralateral kidneys of neonatal mice followed by a strong decrease in *Gdnf* message over time. Accordingly, Gdnf protein expression decreased significantly in the neonatal UUO-kidney indicating the severe disruption of nephron maturation after obstruction. Only intact opposite and sham-operated kidneys of neonatal mice demonstrated an increase in Gdnf expression, likely reflecting the renal developmental stage. Correspondingly, the Gdnf upregulation was not present in kidneys of adult mice with completed nephrogenesis. A combination of Pax2, Eya1, and most importantly Six2 regulate the *Gdnf* gene expression^49^. Our finding that upregulation of *Six2* mRNA was synchronized with *Gdnf* message are in line with those observations. The rapid decline of both genes in neonatal kidneys can be explained by the loss of Six2 during nephrogenesis^50^. Lineage tracing studies have shown that all nephron cell types are formed from the Six2-expressing cap mesenchyme, which is not present in the human postnatal kidney but is still present in the mouse postnatal kidney until nephrogenesis ends^17^. The loss of the Six2 progenitor population therefore restricts the options for regeneration and repair. Nevertheless, the neonatal as well the adult kidney can undergo substantial repair and stimulate compensatory growth in the intact opposite kidney.

Proliferation of tubular and glomerular cells is significantly higher in neonatal than adult kidneys and compensatory renal growth of the contralateral kidney is stronger in neonatal than adult UUO^11^. Accordingly, the calculated kidney weight/body weight ratio of neonatal mice after UUO showed a clear compensatory hypertrophy of the intact opposite kidney in comparison to the sham-operated control. This process of compensatory hypertrophy is regulated by different genes and signaling pathways. In a neonatal UUO model on fetal sheep, numerous protein coding genes could be identified that are differentially regulated following obstruction^8^. In adult UUO models, upregulated genes in the contralateral kidney belong mostly to transporter and membranes families and are associated with metabolism^51^. Our data here demonstrated a possible involvement of *Bmp4* in compensatory renal growth in neonatal mice with UUO. BMP4 expression increased markedly in intact opposite kidneys of neonatal mice but not in adult mice, suggesting a unique BMP4 dependent repair mechanism in the developing kidney with injury. Developmental gene *Bmp4* promotes proliferation in the metanephric mesenchyme and inhibits apoptosis in several tissues^36^. BMP4 has been found to play a crucial role in wound healing response and thymic regeneration^52,53^; it triggers pulmonary vascular remodeling and supports self-renewal of embryonic stem cells^54,55^, emphasizing its importance for regenerative processes. BMP4 is linked to pathological cardiac hypertrophy through activation of proliferation pathways^56,57^; thus, it may also contribute to compensatory renal growth. In our study BMP4 did not co-localize to Ki67 positive proliferating cells in the intact opposite kidney of neonatal mice, suggesting that BMP4 mediates nephron hypertrophy without stimulating proliferation.

To address the question whether compensatory renal growth of the intact opposite kidney is associated with higher proliferation, Ki67 expression was measured in the obstructed, intact opposite and sham-operated kidneys in neonatal mice after UUO. Interestingly, there was no significant difference in the number of Ki67 positive cells between neonatal IO-kidneys and sham-operated controls. Ki67 starts being expressed in the G1 phase, thus it is a marker for cells that have entered the cell cycle^9,58^. It does not differentiate between successful proliferation, resulting in hyperplasia, and cells in cell cycle arrest, leading to hypertrophy. Additionally, measurements of apoptosis did not show significant differences between neonatal IO-kidneys and sham-operated controls. Thus, proliferation and apoptosis do not seem to lead to compensatory growth of the neonatal contralateral kidney. Given this, alternative mechanisms of compensatory renal growth were elaborated.

Compensatory growth is different from normal growth. At a cellular level, it is predominantly characterized by cellular hypertrophy (increased cell size and mass), and less by hyperplasia (increased cell number)^59^. Increased cell size is due to stimulated RNA and protein synthesis, which is regulated by mTORC1 (mammalian target of rapamycin complex 1) signaling. Recently it could be shown that class III phosphatidylinositol-3 kinase (Pik3c3) acts upstream of mTORC1 signaling and mediates protein synthesis and nephron growth in adult mice after completion of nephrogenesis^12^. Pik3c3 is activated by increased amino acid delivery to the remaining kidney due to increased renal blood flow. The expression level of Pik3c3 controls the degree of contralateral renal growth, thus making it a suitable marker for it. Here we show for the first time, that Pik3c3 is upregulated in neonatal kidneys with compensatory hypertrophy. Pik3c3 expression was stronger in adult IO-kidneys compared to neonatal kidneys, emphasizing again the differential regulation in neonatal and adult kidneys after injury^7,12^*.* Even though the upregulation of Pik3c3 expression was less pronounced in neonatal IO-kidneys, it increased over time, in contrast to sham-operated controls. This indicates that protein synthesis, more so than increased proliferation or attenuated apoptosis, represents the driving force behind contralateral renal growth in both adult and neonatal kidneys.

Interaction between BMP4 and the Pik3/Akt pathway has been shown before^60,61^. However, it was not specified which class of Pik3 interacted with BMP4. We would like to speculate that BMP4 may be able to activate Pik3c3 and stimulate compensatory nephron hypertrophy in neonatal mice. Whether BMP4 and Pik3c3 interact in this specific setting needs to be studied in the future.

Together, these data show that certain renal developmental genes are upregulated after unilateral ureteral obstruction, possibly in order to induce regeneration and repair processes. This upregulation is substantially stronger and faster in neonatal than in adult kidneys. While neonatal kidneys directly stimulate their developmental program, adult kidneys only show partial and weaker upregulation after persisting damage. This implies that regenerative and repair processes are differentially regulated in neonatal and adult kidneys. Most of the genes in this study promote proliferation and inhibit apoptosis. They may contribute to compensatory growth processes in the contralateral kidney. However, increased proliferation and attenuated apoptosis appear to have only a minor influence on compensatory renal growth in the neonatal kidney. Protein synthesis seems to be the driving force behind the compensatory growth of the intact opposite kidney. This is mediated by Pik3c3, which is activated through increased renal blood flow in the contralateral kidney of neonatal and adult mice.

## Supplementary information

Supplementary Information.
