# Supplementary material for: Renal developmental genes are differentially regulated after unilateral ureteral obstruction in neonatal and adult mice
Source: Sci Rep. 2020 Nov 9;10:19302. doi: 10.1038/s41598-020-76328-3 (PMC7653944; doi:10.1038/s41598-020-76328-3)
Supplement: Supplementary file 1 — Supplementary Information. [file 41598_2020_76328_MOESM1_ESM.pdf]

Supplementary Material

Renal developmental genes are differentially regulated after unilateral ureteral obstruction in neonatal and adult mice

Melanie J. Kubik[1#]; Maja Wyczanska[2#]; Mojca Gasparitsch[2]; Ursula Keller[2]; Stefanie Weber[3]; Franz Schaefer[1]; Bärbel Lange-Sperandio[2]

[1]Department of Pediatrics, Ruprecht-Karls-University, Heidelberg, Germany

[2]Department of Pediatrics, Dr. v. Hauner Children's Hospital, Ludwig-Maximilians-University, Munich, Germany

[3]University Children's Hospital, Philipps-University, Marburg, Germany

# These authors contributed equally to this work

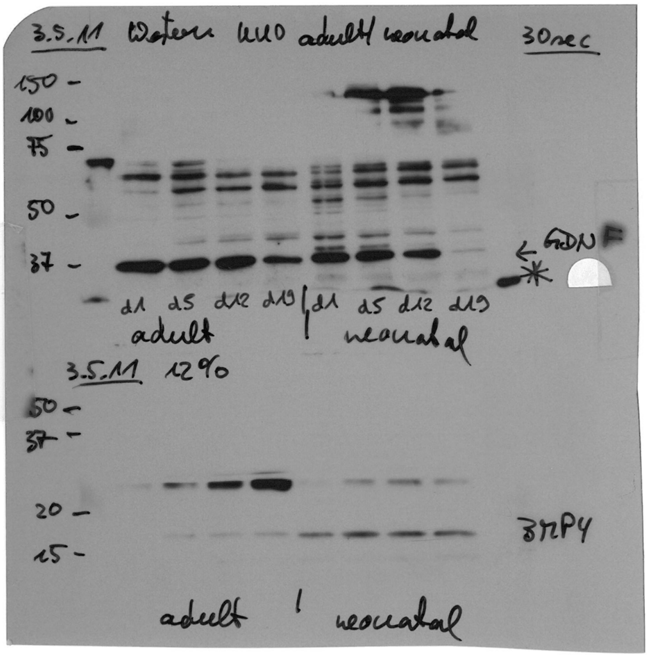

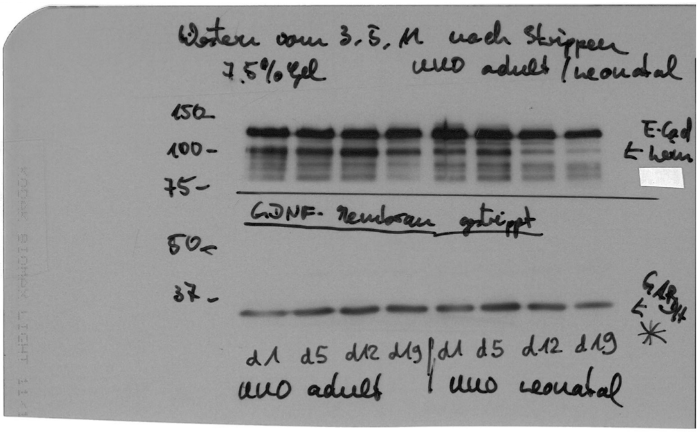

Supplementary Figure S1

Western blot images for GDNF and GAPDH in UUO kidneys. Uncropped western blots for GDNF (32 kDa) and GAPDH (37 kDa) in adult and neonatal obstructed (UUO) kidneys (1, 5, 12, 19 days after obstruction). * marks the sections used in Figure 3. GDNF and GAPDH were visualized separately because of similar sizes, but they represent the same gel.

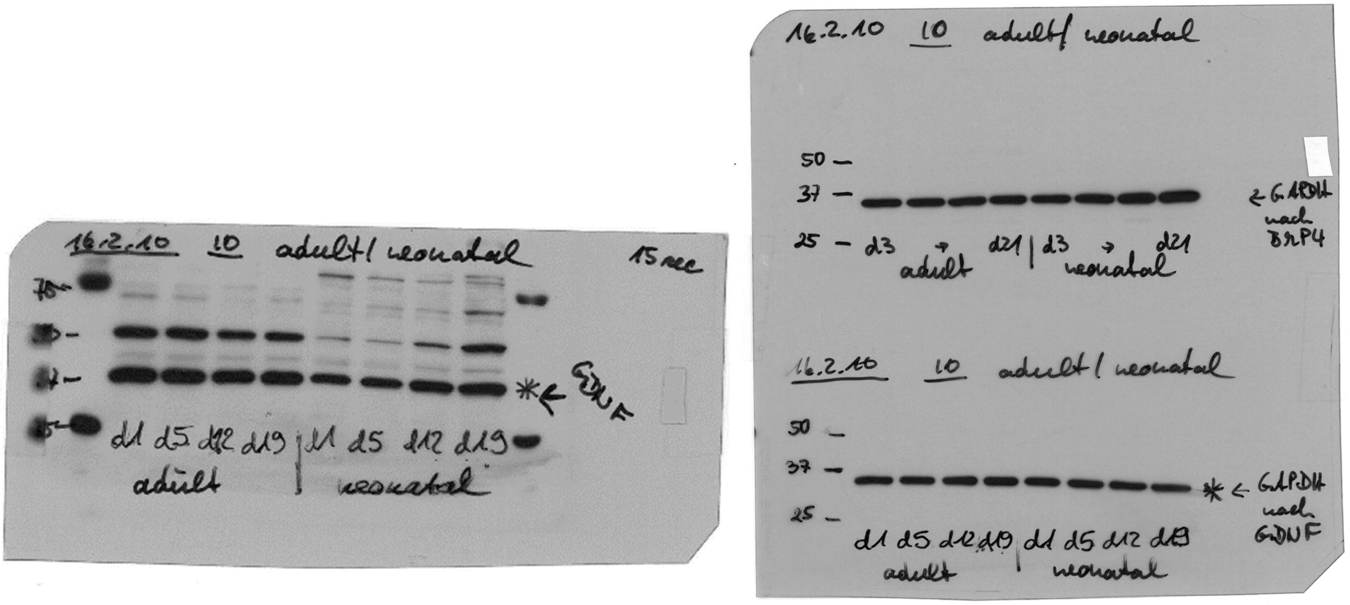

Supplementary Figure S2

Western blot images for GDNF and GAPDH in IO kidneys. Uncropped western blots for GDNF (32 kDa) and GAPDH (37 kDa) in adult and neonatal intact opposite (IO) kidneys (1, 5, 12, 19 days after obstruction). * marks the sections used in Figure 3. GDNF and GAPDH were visualized separately because of similar sizes, but they represent the same gel.

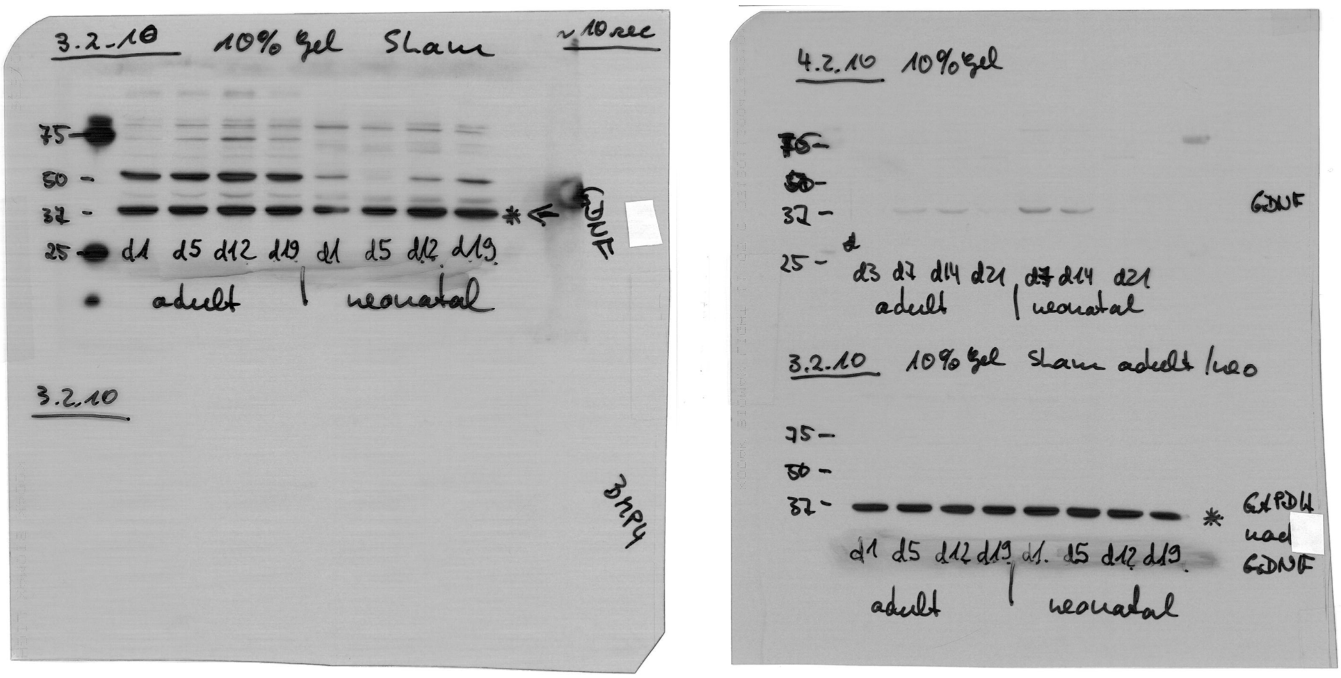

Supplementary Figure S3

Western blot images for GDNF and GAPDH in sham-operated kidneys. Uncropped western blots for GDNF (32 kDa) and GAPDH (37 kDa) in adult and neonatal sham-operated (Sham) kidneys (1, 5, 12, 19 days after sham operation). * marks the sections used in Figure 3. GDNF and GAPDH were visualized separately because of similar sizes, but they represent the same gel.

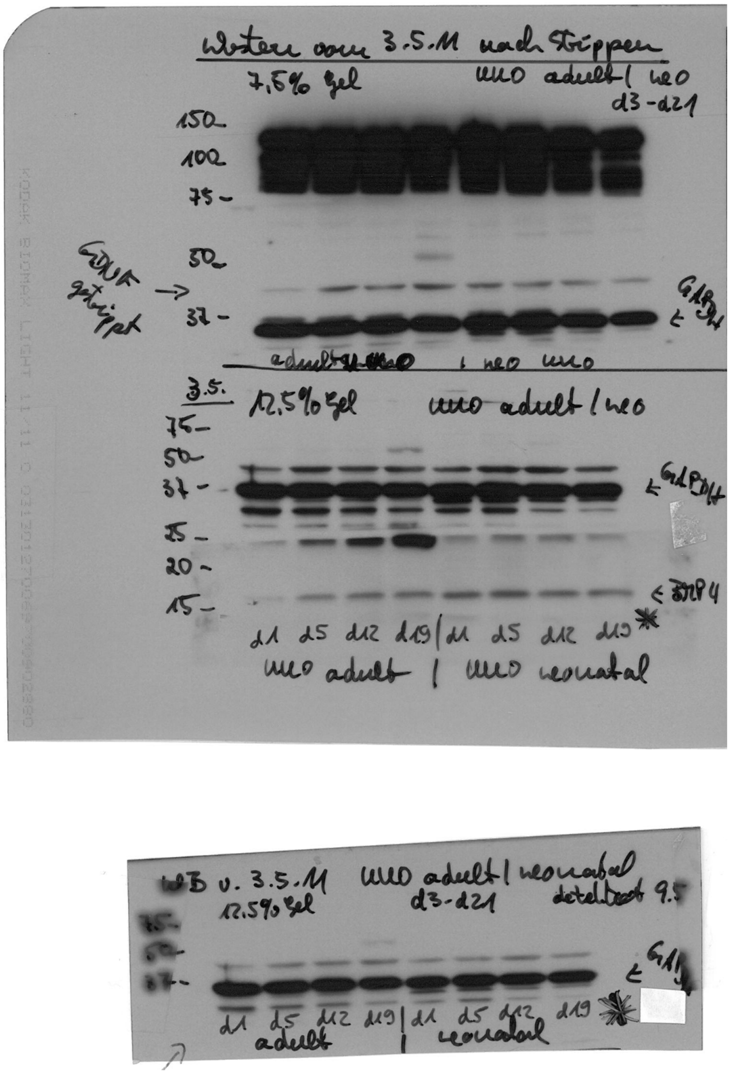

Supplementary Figure S4

Western blot images for BMP4 and GAPDH in UUO kidneys. Uncropped western blots for BMP4 (15 kDa) and GAPDH (37 kDa) in adult and neonatal obstructed kidneys (UUO) (1, 5, 12, 19 days after obstruction). * marks the sections used in Figure 3. BMP4 and GAPDH were visualized separately because of different film exposer times, but they represent the same gel.

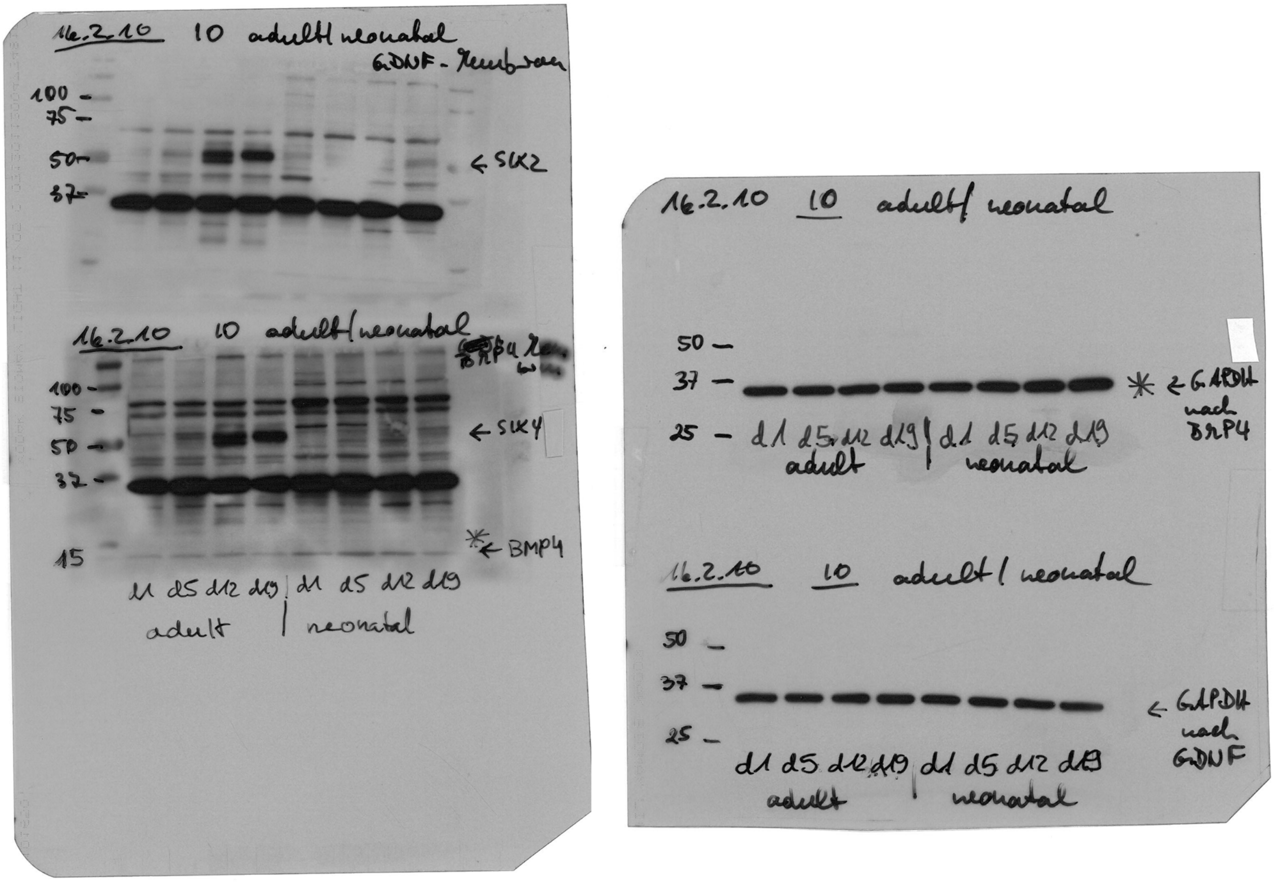

Supplementary Figure S5

Western blot images for BMP4 and GAPDH in IO kidneys. Uncropped western blots for BMP4 (15 kDa) and GAPDH (37 kDa) in adult and neonatal intact opposite (IO) kidneys (1, 5, 12, 19 days after obstruction). * marks the sections used in Figure 3. BMP4 and GAPDH were visualized separately because of different film exposer times, but they represent the same gel.

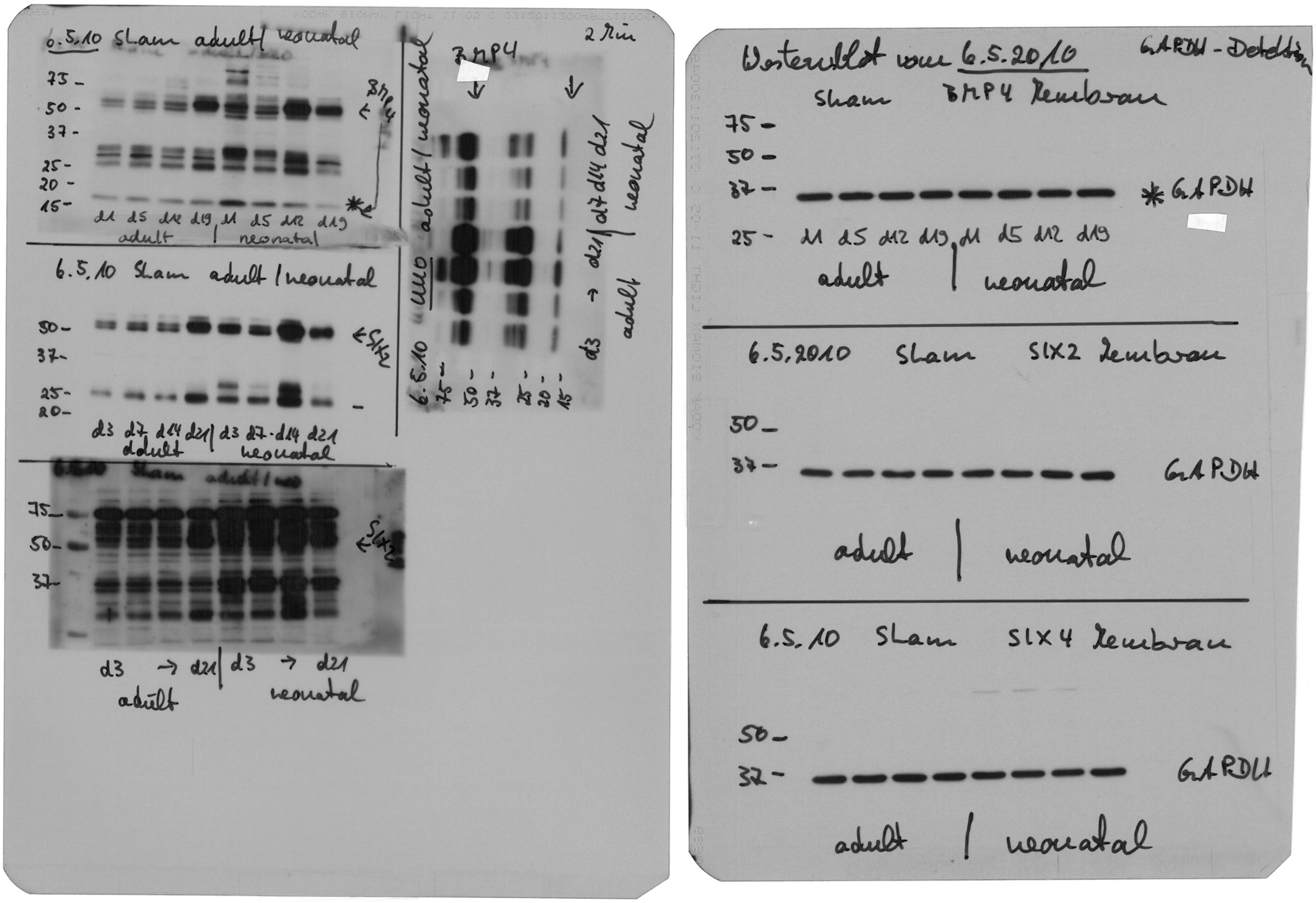

Supplementary Figure S6

Western blot images for BMP4 and GAPDH in sham-operated kidneys. Uncropped western blots for BMP4 (15 kDa) and GAPDH (37 kDa) in adult and neonatal sham-operated (Sham) kidneys (1, 5, 12, 19 days after sham operation). * marks the sections used in Figure 3. BMP4 and GAPDH were visualized separately because of different film exposer times, but they represent the same gel.

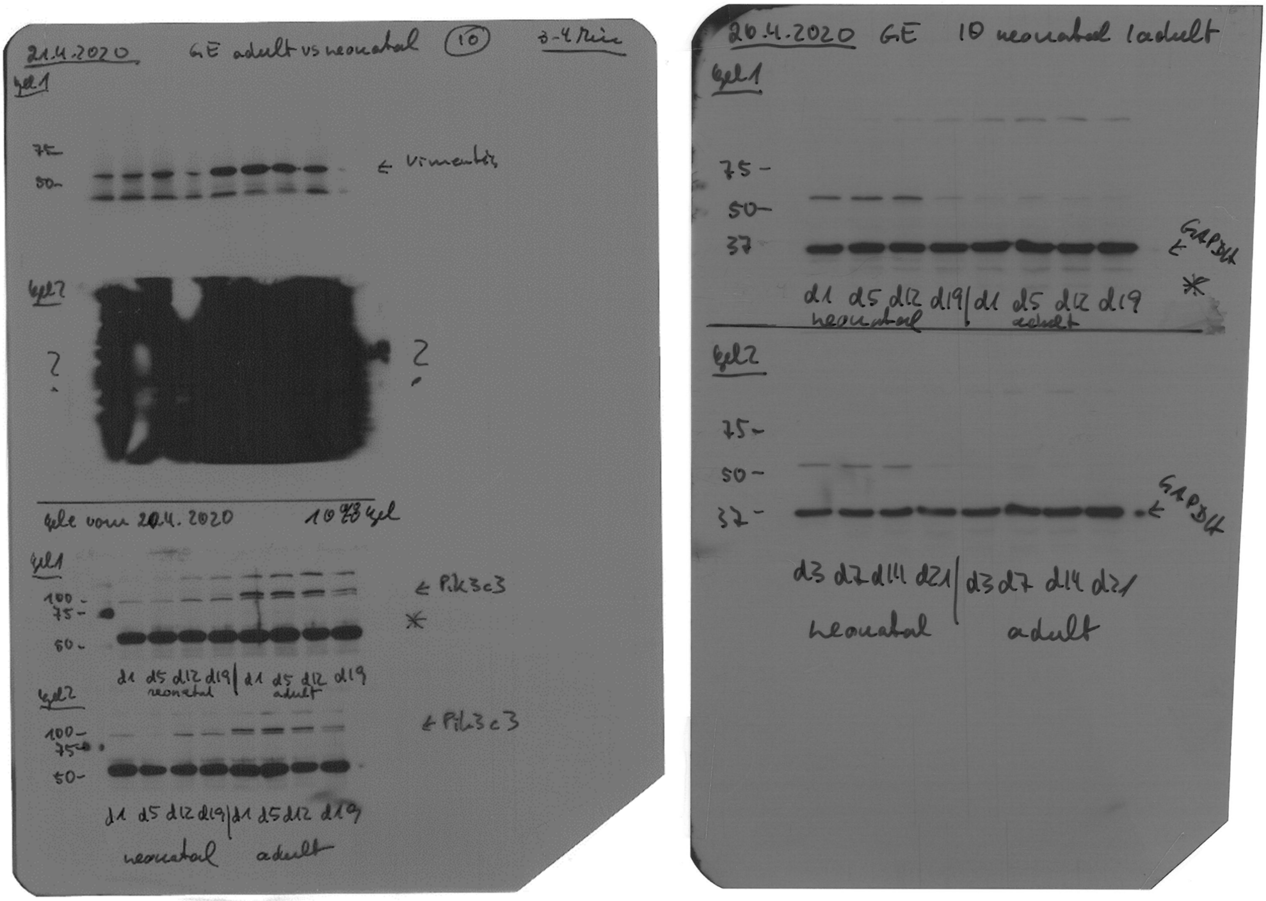

Supplementary Figure S7

Western blot images for Pik3c3 and GAPDH in IO kidneys. Uncropped western blots for Pik3c3 (100 kDa) and GAPDH (37 kDa) in adult and neonatal intact opposite (IO) kidneys (1, 5, 12, 19 days after obstruction). * marks the sections used in Figure 7. Pik3c3 and GAPDH were visualized separately because of different film exposer times, but they represent the same gel.

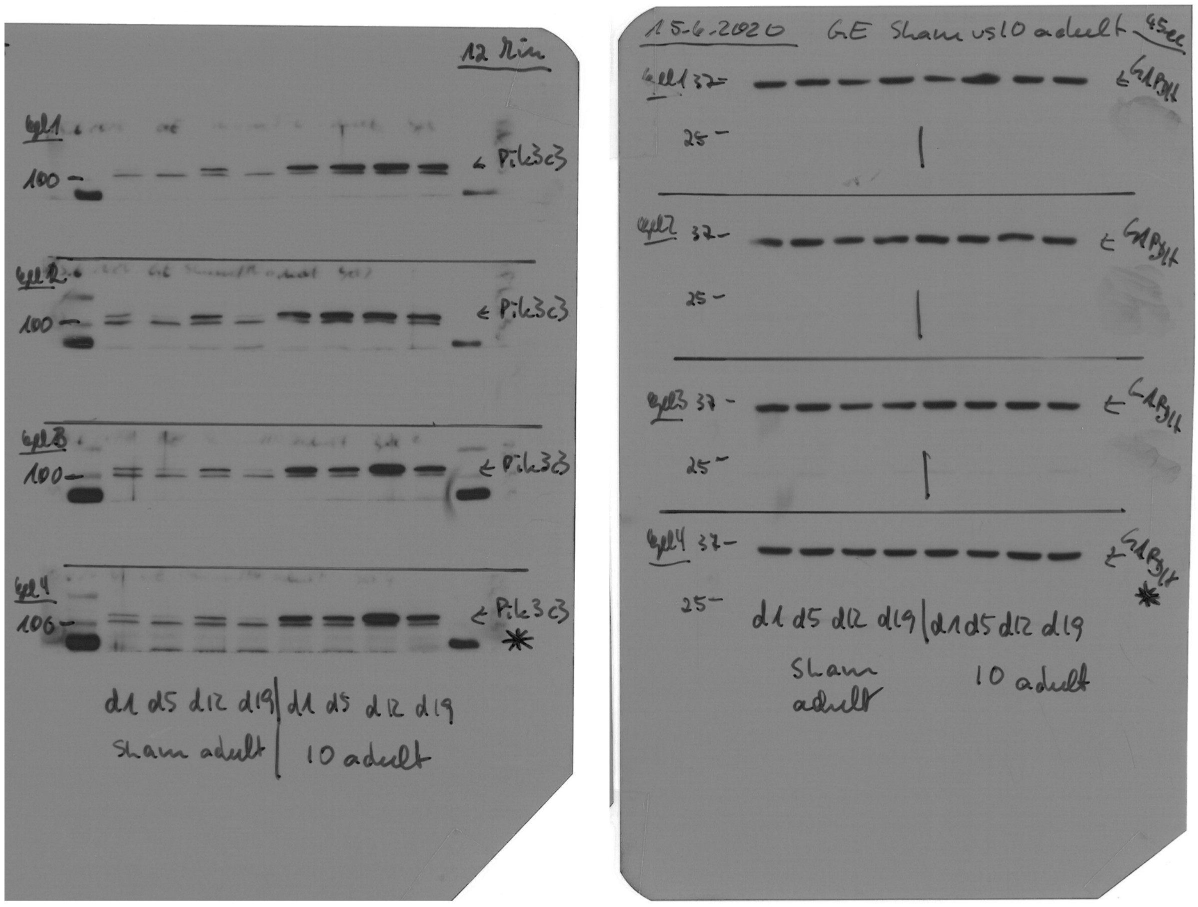

Supplementary Figure S8

Western blot images for Pik3c3 and GAPDH in adult kidneys. Uncropped western blots for Pik3c3 (100 kDa) and GAPDH (37 kDa) in adult sham-operated (Sham) intact opposite (IO) kidneys (1, 5, 12, 19 days after obstruction). * marks the sections used in Figure 7. Pik3c3 and GAPDH were visualized separately because of different film exposer times, but they represent the same gel.

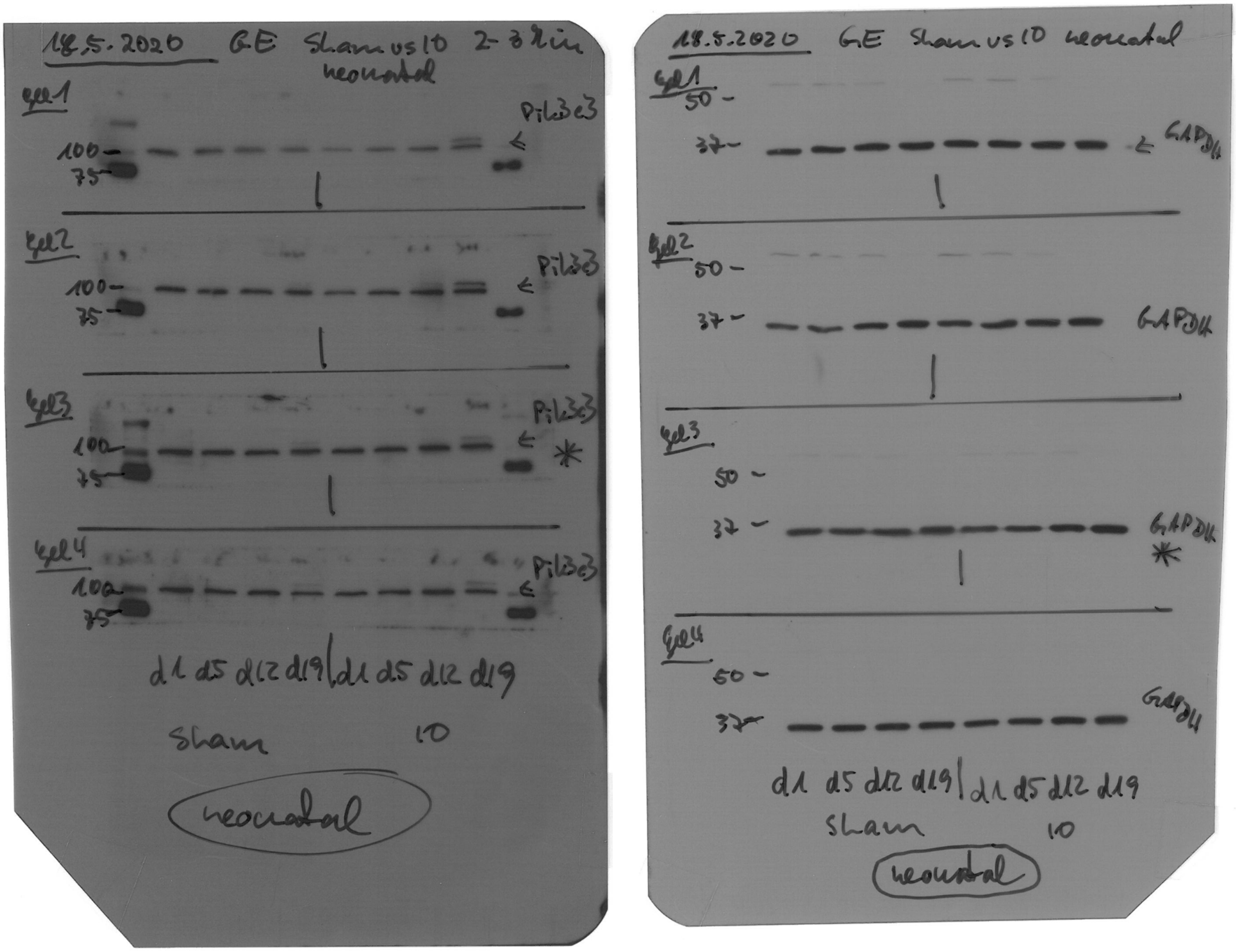

Supplementary Figure S9

Western blot images for Pik3c3 and GAPDH in neonatal kidneys. Uncropped western blots for Pik3c3 (100 kDa) and GAPDH (37 kDa) in neonatal sham-operated (Sham) intact opposite (IO) kidneys (1, 5, 12, 19 days after obstruction). * marks the sections used in Figure 7. Pik3c3 and GAPDH were visualized separately because of different film exposer times, but they represent the same gel.

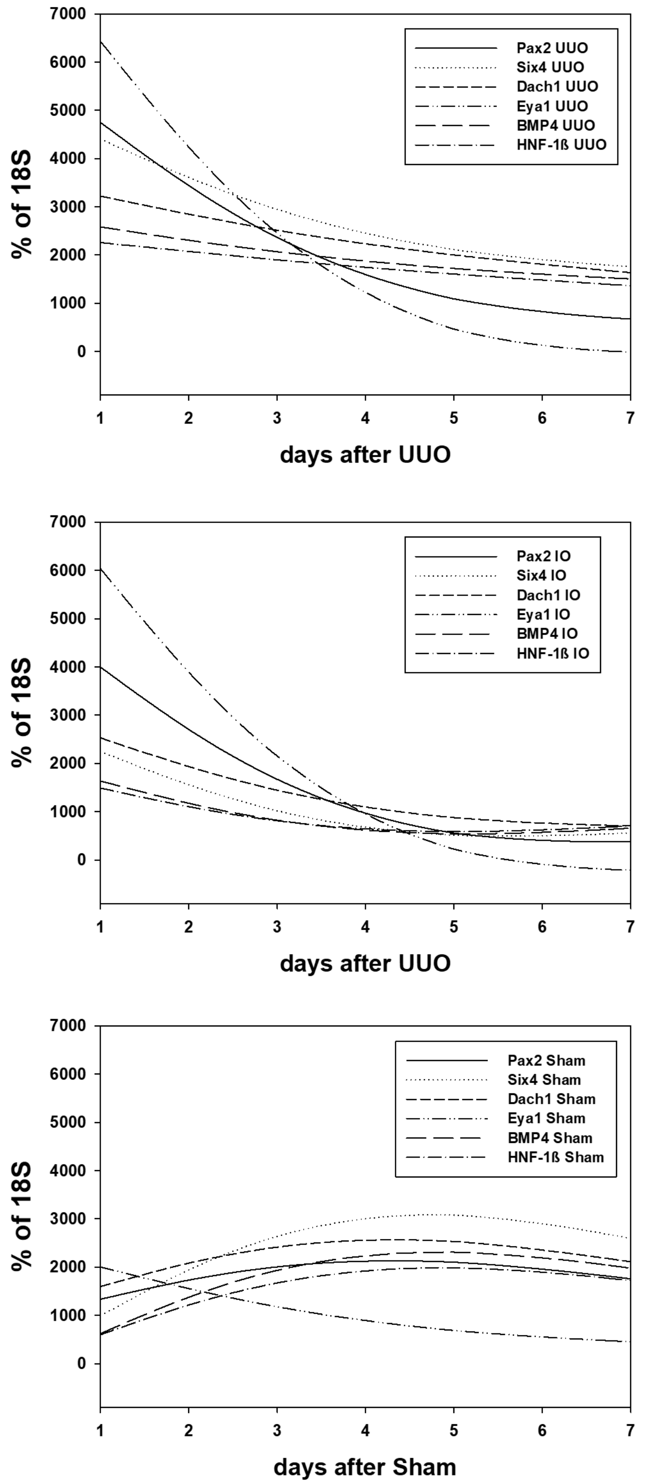

Supplementary figure S10

Gene expression of renal developmental genes in neonatal kidneys in the first 5 days after surgery. Gene expression of *Pax2, Six4, Dach1, Eya1, Bmp4, Hnf-1ß* was analyzed in neonatal UUO-kidneys, intact opposite (IO) kidneys and in sham-operated controls of WT mice 1-5 days after UUO. Unilateral ureteral obstruction or sham operation was performed in neonatal mice on the second day of life.

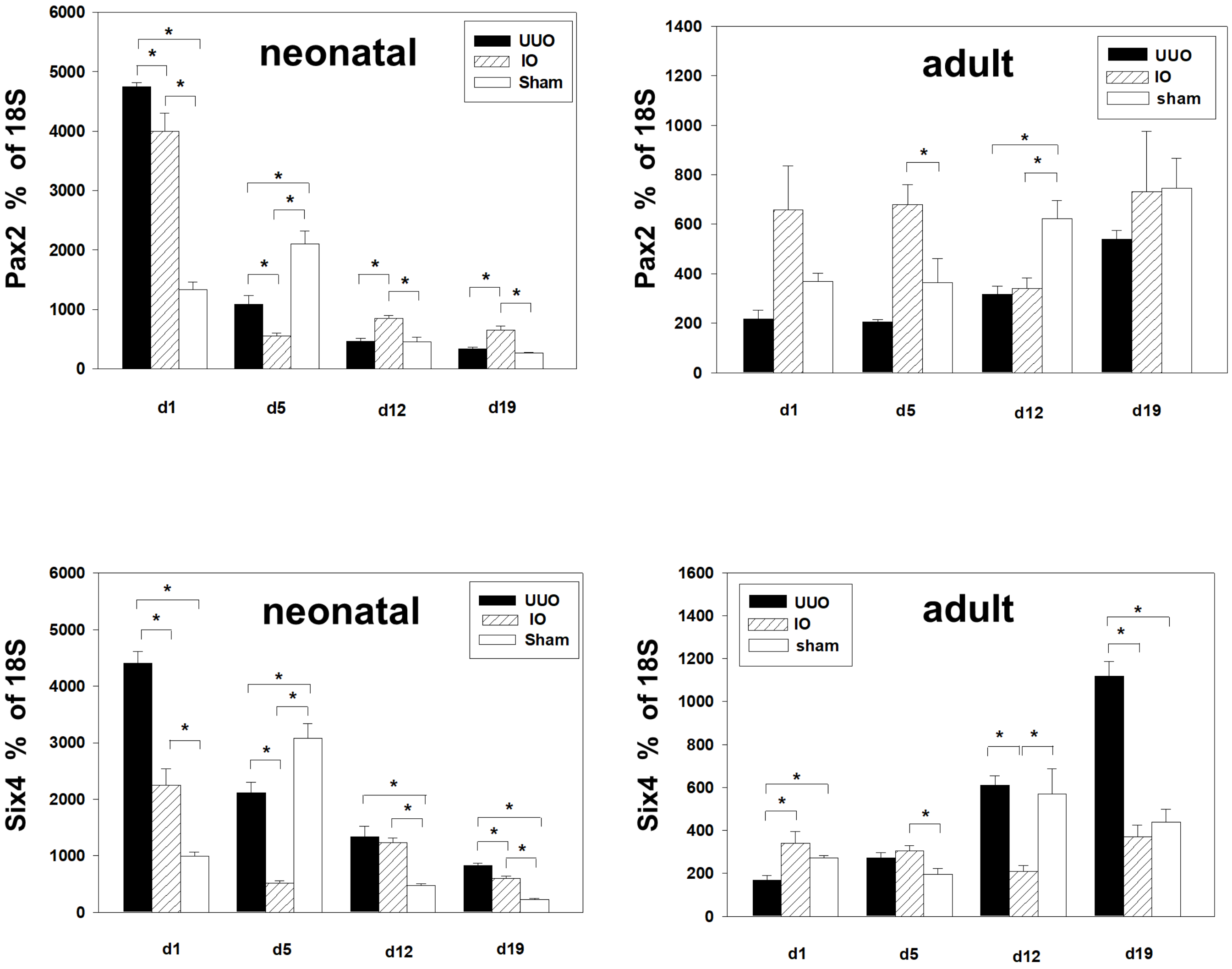

Supplementary figure S11

Gene expression of *Pax2* and *Six4* in neonatal and adult kidneys. Quantitative RT-PCR of renal developmental genes in neonatal and adult kidneys of WT mice 1, 5, 12, and 19 days after UUO. Surgery was performed in neonatal mice on the second day of life and in adult mice at the age of 7-8 weeks. UUO = unilateral ureteral obstruction, IO = intact opposite kidney, sham= sham operated control kidney. * $p<0.05$

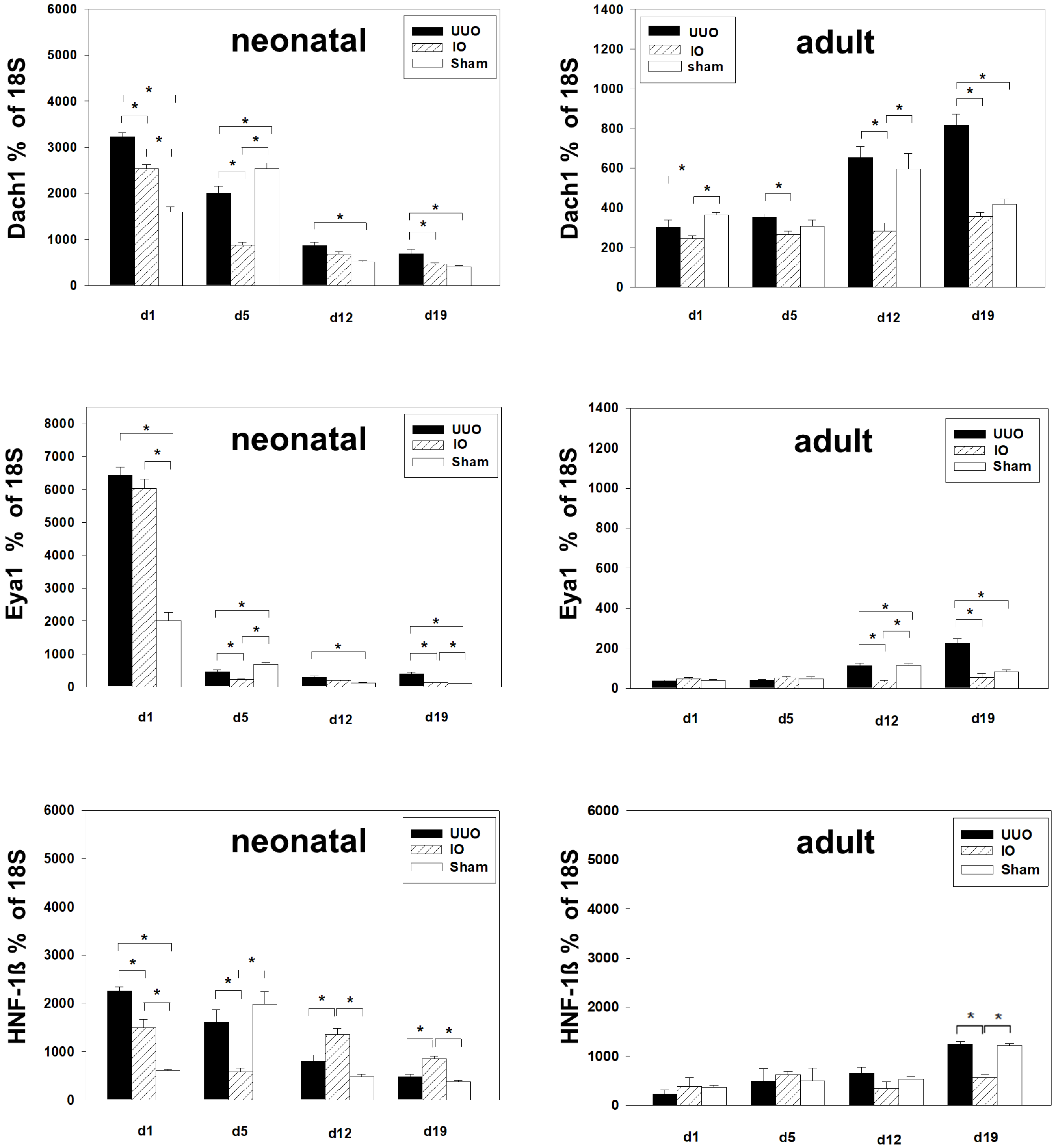

Supplementary figure S12

Gene expression of *Dach1*, *Eya1* and *HNF-1β* in neonatal and adult kidneys. Quantitative RT-PCR of renal developmental genes in neonatal and adult kidneys of WT mice 1, 5, 12, and 19 days after UUO. Surgery was performed in neonatal mice on the second day of life and in adult mice at the age of 7-8 weeks. UUO = unilateral ureteral obstruction, IO = intact opposite kidney, sham= sham operated control kidney. * $p<0.05$

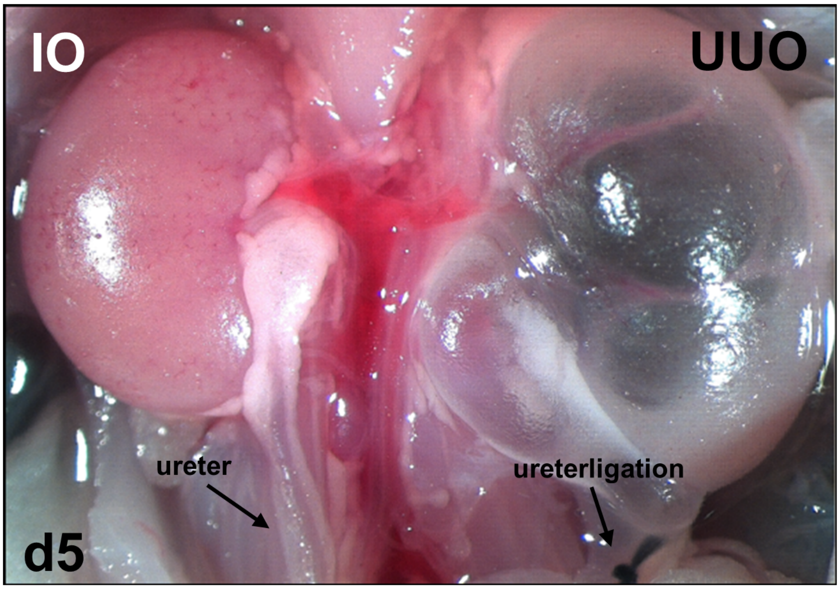

Supplementary figure S13

Photograph of a neonatal kidney after unilateral ureteral obstruction. Neonatal kidney at day 5 after surgery. Surgery was performed in neonatal mice on the second day of life. IO = intact opposite kidney, UUO = unilateral ureteral obstruction.

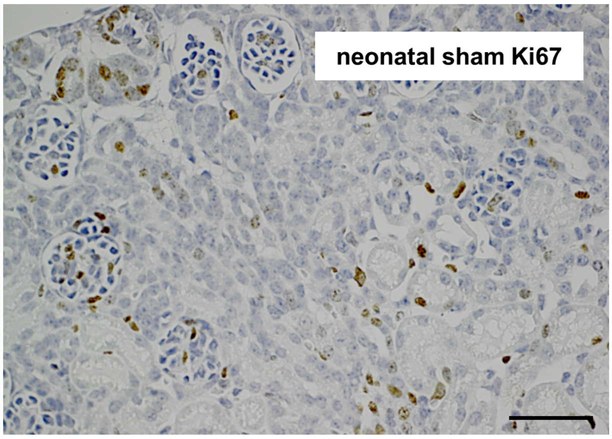

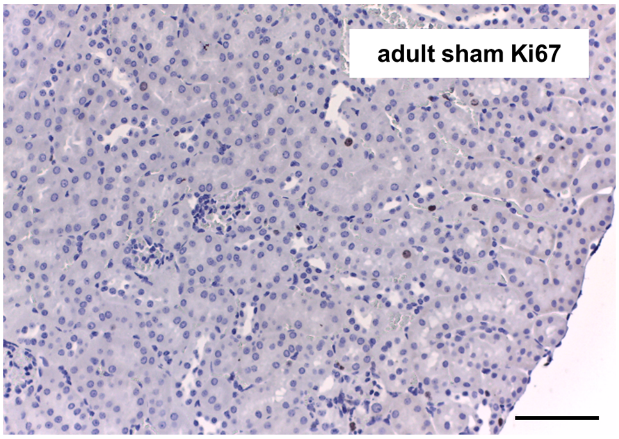

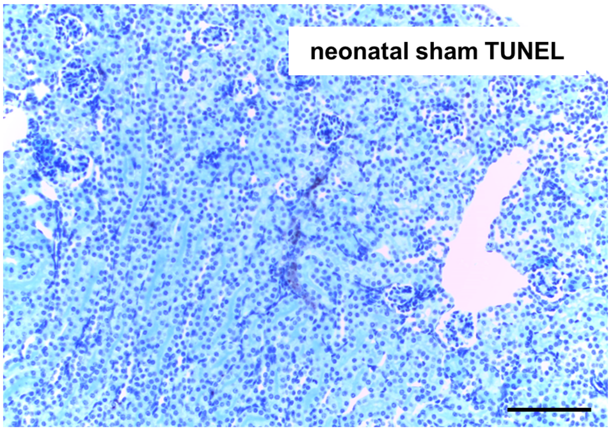

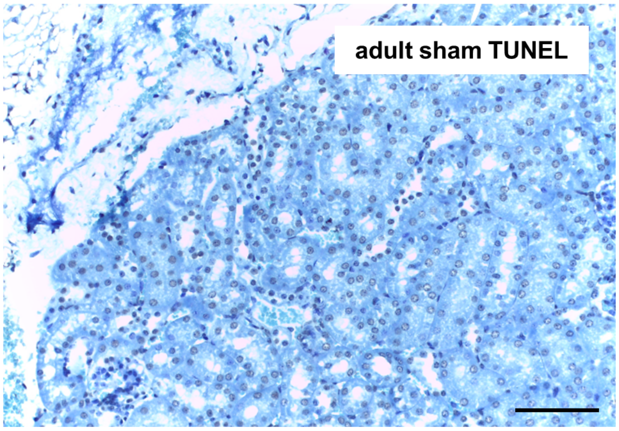

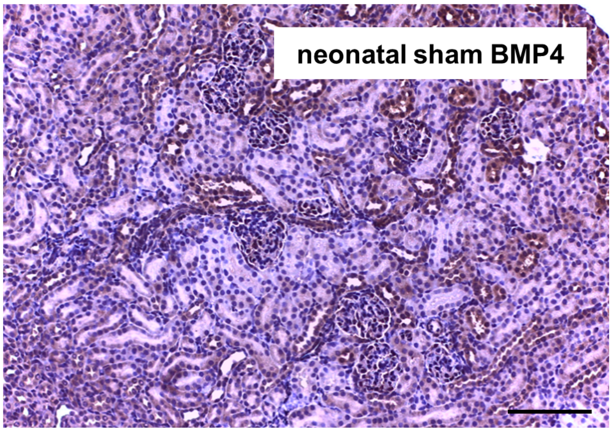

Supplementary figure S14

Immunohistochemical staining of Ki67, TUNEL and BMP4 in sham-operated kidneys. Immunohistochemical staining of Ki67 (proliferation), TUNEL (apoptosis) and BMP4 in neonatal and adult sham-operated kidneys. Magnification of 200x. Bar = 100 µm

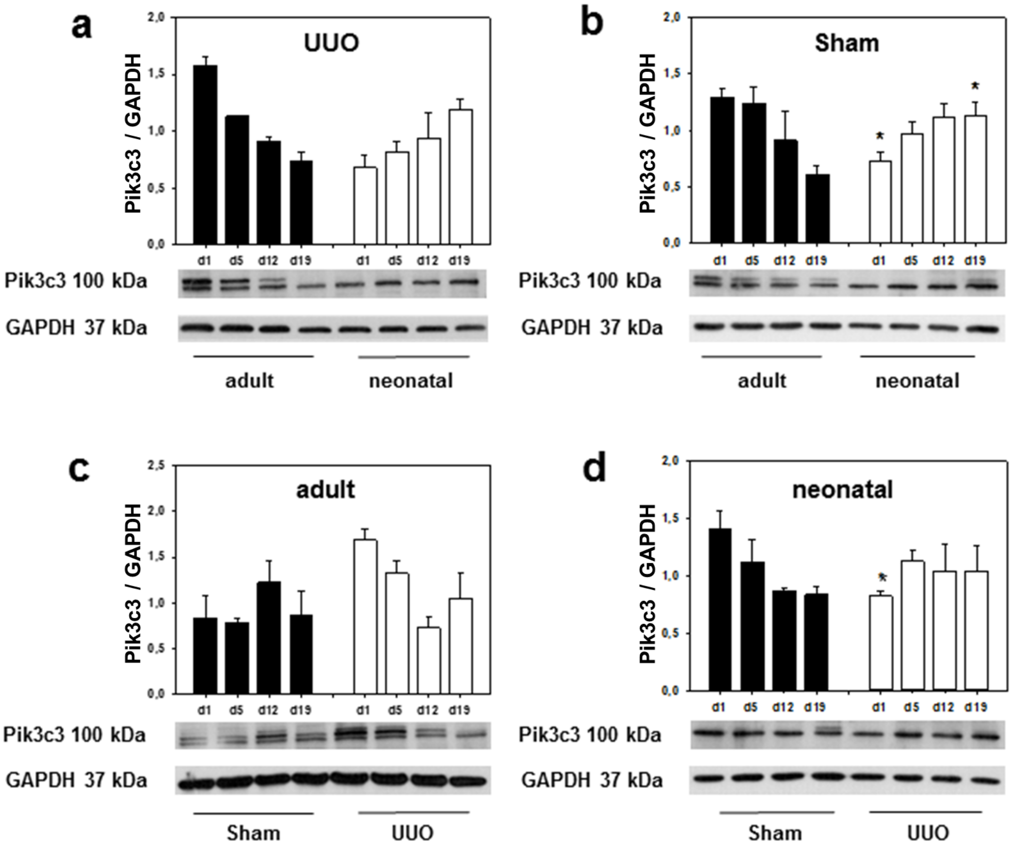

Supplementary figure S15

Phosphatidylinositol 3 kinase class III (Pik3c3) expression. Neonatal and adult mice were subjected to UUO or sham operation. Whole kidneys were processed for western blot analysis at day 1, 5, 12, and 19 after surgery. n=3; *$p<0.05$ Significance markings reflect the differences between groups, compared to each other on the same time points. The brightness of the western blot images was changed after the analysis for uniform presentation. The shown western blot images are cropped, for uncropped western blots see Supplementary Fig. S15-18.

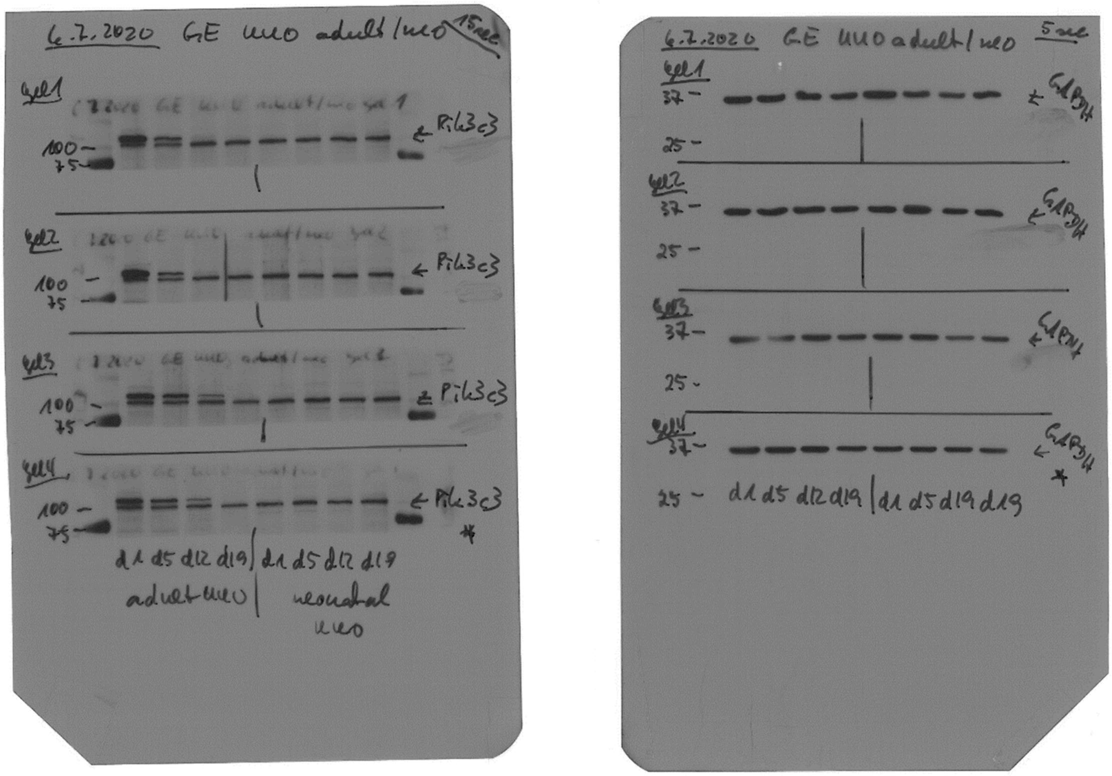

Supplementary figure S16

Western blot images for Pik3c3 and GAPDH in UUO kidneys. Uncropped western blots for Pik3c3 (100 kDa) and GAPDH (37 kDa) in adult and neonatal obstructed (UUO) kidneys (1, 5, 12, 19 days after obstruction). * marks the sections used in Supplementary Figure S15. Pik3c3 and GAPDH were visualized separately because of different film exposer times, but they represent the same gel.

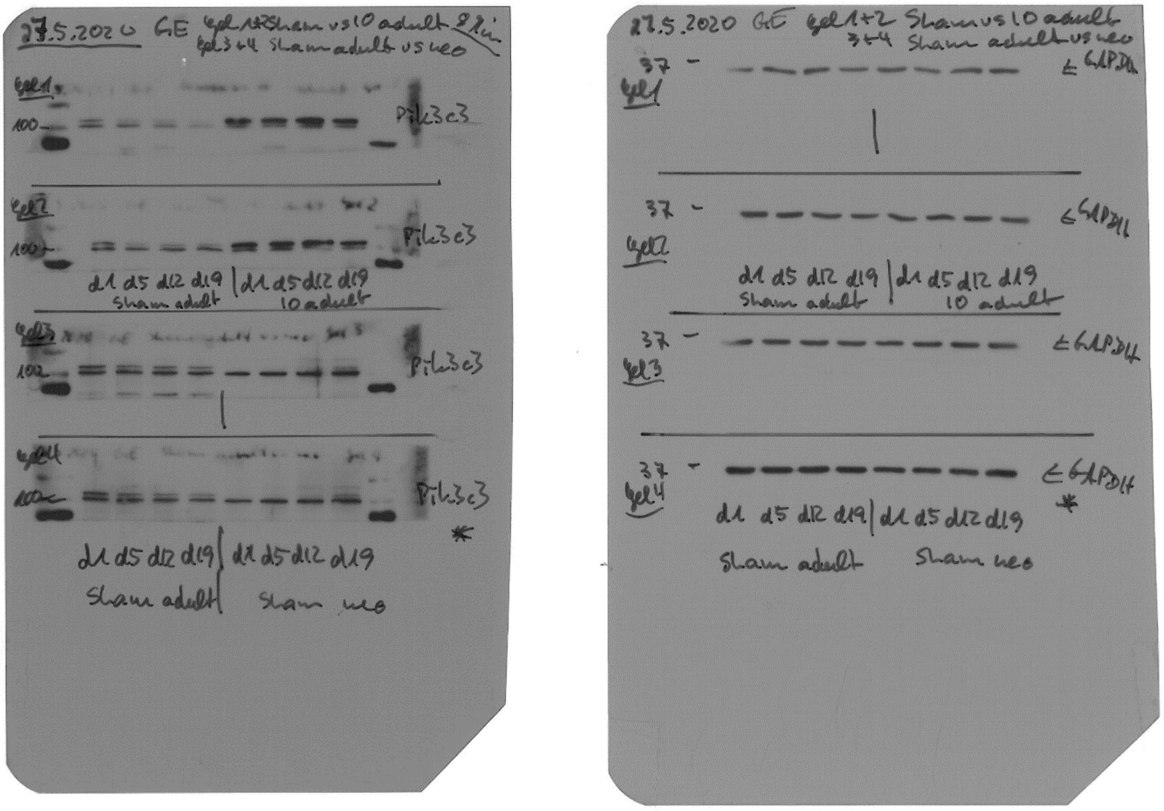

Supplementary figure S17

Western blot images for Pik3c3 and GAPDH in sham-operated kidneys. Uncropped western blots for Pik3c3 (100 kDa) and GAPDH (37 kDa) in adult and neonatal sham-operated kidneys (1, 5, 12, 19 days after obstruction). * marks the sections used in Supplementary Figure S15. Pik3c3 and GAPDH were visualized separately because of different film exposer times, but they represent the same gel.

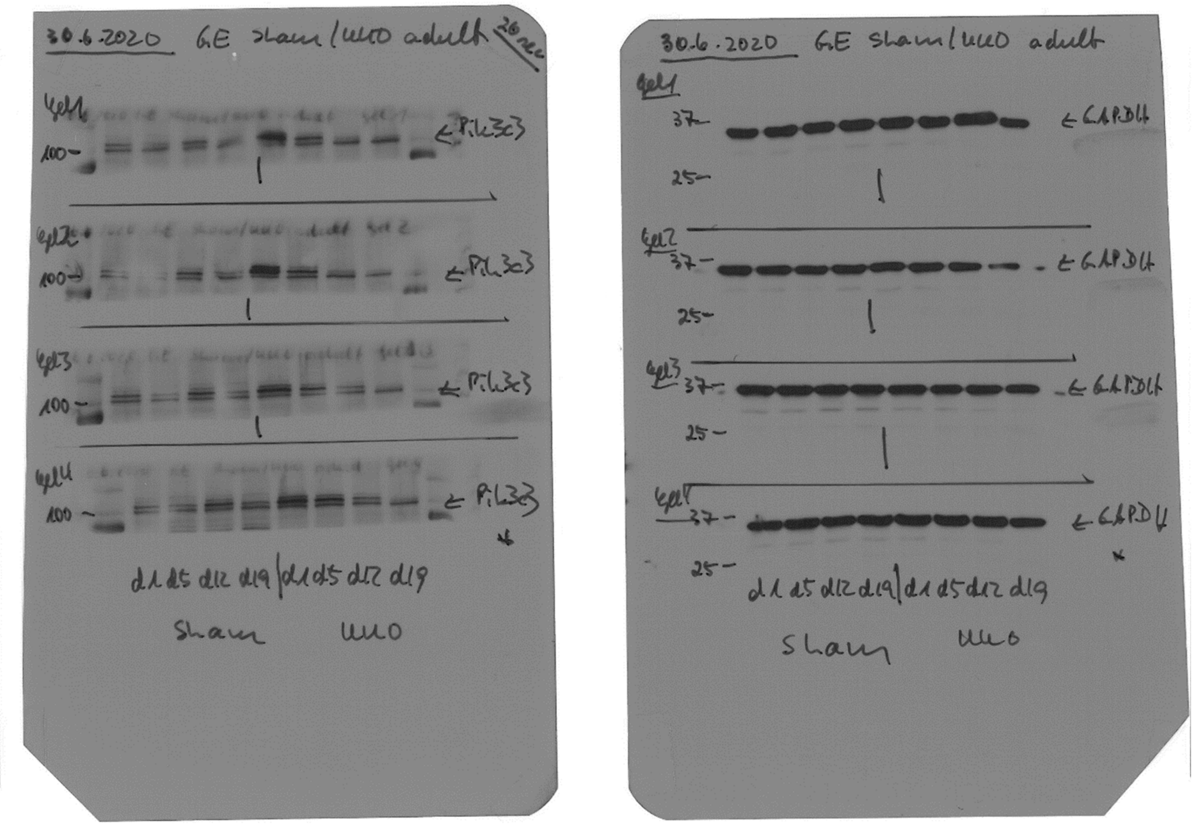

Supplementary figure S18

Western blot images for Pik3c3 and GAPDH in adult kidneys. Uncropped western blots for Pik3c3 (100 kDa) and GAPDH (37 kDa) in obstructed (UUO) and sham-operated adult kidneys (1, 5, 12, 19 days after obstruction). * marks the sections used in Supplementary Figure S15. Pik3c3 and GAPDH were visualized separately because of different film exposer times, but they represent the same gel.

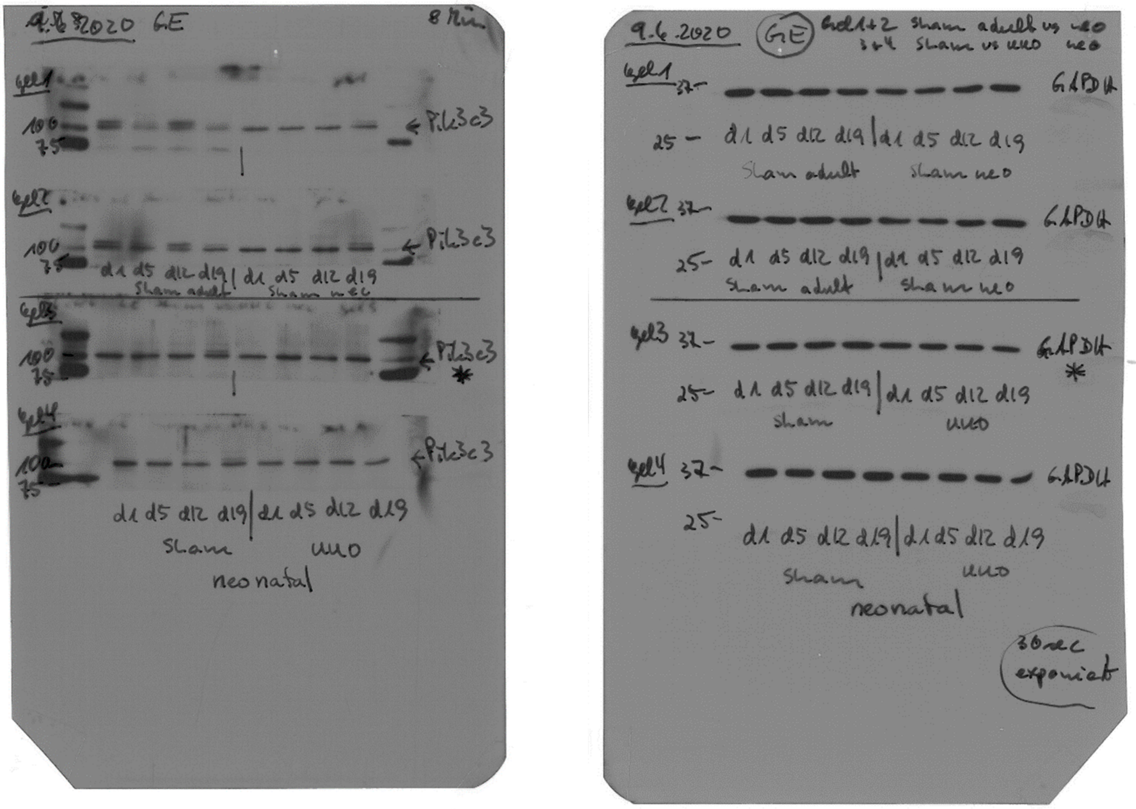

Supplementary figure S19

Western blot images for Pik3c3 and GAPDH in neonatal kidneys. Uncropped western blots for Pik3c3 (100 kDa) and GAPDH (37 kDa) in obstructed (UUO) and sham-operated neonatal kidneys (1, 5, 12, 19 days after obstruction). * marks the sections used in Supplementary Figure S15. Pik3c3 and GAPDH were visualized separately because of different film exposer times, but they represent the same gel.

Supplementary Table S1. GDNF and BMP4 expression

			**GDNF**	**BMP4**
Group	Comparison	Days after obstruction/sham	P value	P value
neonatal	UUO vs Sham	1	n.s.	n.s.
		5	n.s.	n.s.
		12	0.016	n.s.
		19	0.025	n.s.
	IO vs Sham	1	n.s.	n.s.
		5	n.s.	n.s.
		12	n.s.	0.005
		19	n.s.	0.011
adult	UUO vs Sham	1	n.a.	n.a.
		5	n.a.	n.a.
		12	n.a.	n.a.
		19	n.a.	n.a.
	IO vs Sham	1	n.s.	n.s.
		5	n.s.	n.s.
		12	n.s.	n.s.
		19	0.011	n.s.
UUO	neonatal vs. adult	1	0.023	0.028
		5	0.004	n.s.
		12	n.s.	n.s.
		19	n.s.	n.s.
IO	neonatal vs. adult	1	0.032	n.s.
		5	n.s.	0.011
		12	n.s.	<0.001
		19	n.s.	0.003
Sham	neonatal vs. adult	1	n.s.	0.004
		5	0.022	0.028
		12	n.s.	0.003
		19	n.s.	n.s.

Neonatal and adult mice were subjected to UUO or sham operation. Whole kidneys were processed for western blot analysis at day 1, 5, 12, and 19 after surgery. $p < 0.05$; n.s. = not significant; n.a. = not applicable

Supplementary Table S2. Proliferation and apoptosis

**Proliferation**				
Compartments	Days after UUO/Sham	P value UUO vs IO	P value UUO vs Sham	P value IO vs Sham
tubular	d1	<0.001	<0.001	n.s.
	d5	<0.001	<0.001	n.s.
	d12	0.050	0.022	n.s.
	d19	<0.001	<0.001	n.s.
interstitial	d1	<0.001	<0.001	n.s.
	d5	0.038	0.001	n.s.
	d12	<0.001	<0.001	n.s.
	d19	0,004	<0.001	n.s.
glomerular	d1	<0.001	<0.001	0.001
	d5	<0.001	<0.001	n.s.
	d12	<0.001	<0.001	n.s.
	d19	n.s.	n.s.	n.s.
**Apoptosis**				
tubular	d1	n.s.	n.s.	n.s.
	d5	<0.001	<0.001	n.s.
	d12	<0.001	<0.001	n.s.
	d19	<0.001	<0.001	n.s.
interstitial	d1	n.s.	n.s.	n.s.
	d5	<0.001	<0.001	n.s.
	d12	<0.001	<0.001	n.s.
	d19	<0.001	<0.001	n.s.
glomerular	d1	n.s.	n.s.	n.s.
	d5	n.s.	n.s.	n.s.
	d12	<0.001	n.s.	0.008
	d19	0.009	n.s.	0.002

Renal sections of UUO-, IO- and sham-operated kidneys of neonatal mice were stained for tubular, interstitial and glomerular proliferation (Ki67 antibody) and apoptosis (TUNEL) at day 1, 5, 12, and 19 after surgery. $p < 0.05$; n.s. = not significant

Supplementary Table S3. Pik3c3 expression

**Pik3c3**			
Group	Comparison	Days after obstruction/sham	P value
neonatal	UUO vs sham	1	0.022
		5	n.s.
		12	n.s.
		19	n.s.
	IO vs sham	1	0.002
		5	n.s.
		12	0.024
		19	0.029
adult	UUO vs sham	1	n.a.
		5	n.a.
		12	n.a.
		19	n.a.
	IO vs sham	1	0.023
		5	<0.001
		12	0.004
		19	<0.001
UUO	neonatal vs. adult	1	n.a.
		5	n.a.
		12	n.a.
		19	n.a.
IO	neonatal vs. adult	1	0.01
		5	0.013
		12	0.004
		19	n.s.
sham	neonatal vs. adult	1	0.006
		5	n.s.
		12	n.s.
		19	0.022

Neonatal and adult mice were subjected to UUO or sham operation. Whole kidneys were processed for western blot analysis at day 1, 5, 12, and 19 after surgery. $p < 0.05$; n.s. = not significant; n.a. = not applicable
